# Direct observation of importin α family member KPNA1 in axonal transport with or without a schizophrenia-related mutation

**DOI:** 10.1016/j.jbc.2025.108343

**Published:** 2025-02-24

**Authors:** Katsutoshi Mizuno, Masaki Sugahara, Osamu Kutomi, Ryota Kato, Takafumi Itoh, Satoshi Fujita, Masami Yamada

**Affiliations:** 1 Department of Cell Biology and Biochemistry, Division of Medicine, Faculty of Medical Sciences, University of Fukui, Yoshida-gun, Fukui Prefecture, Japan; 2 Life Science Innovation Center, University of Fukui, Fukui City, Fukui Prefecture, Japan; 3 Department of Frontier Fiber Technology and Science, Graduate School of Engineering, University of Fukui, Fukui City, Fukui Prefecture, Japan; 4 Department of Bioscience and Biotechnology, Fukui Prefectural University, Yoshida-gun, Fukui Prefecture, Japan

**Keywords:** cytoplasmic dynein, kinesin, microtubule, molecular motors, endosome, axonal transport, nuclear transport factors, fluorescence recovery after photobleaching (FRAP), proximity ligation assay (PLA), proteomics, schizophrenia

## Abstract

Karyopherin α1 (KPNA1)/(human importin α5; mouse importin α1) facilitates cargo transport into the nucleus by forming a complex with a nuclear localization sequence containing cargo and importin β1 (IPOB1). The elevated KPNA1 expression in neurons and the correlation between mutations and psychiatric disorders suggest its broader significance beyond nucleocytoplasmic transport. Although KPNA1 is localized in the neurites of neurons, its role in axonal transport mechanisms remains unclear, and data on the connection between psychiatric disorders and signaling at the periphery of neurons remain limited. To address this knowledge gap, we investigated the dynamics of KPNA1 and related factors within axons. Our results showed that many of the axonal KPNA1 did not form a complex with IPOB1 in noninjured steady-state neurons. Axonal KPNA1 exhibited relatively stationary mobility and some showed bidirectional motility with fluctuating motion. KPNA1 partly comigrated with endosome/lysosome-associated factors, suggesting the presence of novel mechanisms underlie axonal transport and nucleocytoplasmic shuttling involving KPNA1 and IPOB1. Mutated KPNA1, which has been shown to be associated with psychiatric disorders (KPNA1^E448X^), was predominantly localized to the nucleus and lost from the axon. Incorporating a nuclear export signal (KPNA1^E448X-NES^) enhanced its subcellular localization and dynamics in the axon. Our findings demonstrate that KPNA1 functions not only as a shuttle between the cytoplasm and nucleus but also as a transporter in neuronal axons, relying on the endosomes for movement away from the nucleus with relatively slow net motions. Furthermore, a mutation in the *Kpna1* gene can affect the dynamics of axonal transport. The insights from these mutations provide valuable knowledge for expanding our understanding of psychiatric disorders and facilitate the development of novel treatment strategies.

Karyopherin αs (KPNAs), also known as importin α, are nuclear import factors that shuttle between the cytoplasm and nucleus, facilitating interactions between cargo-containing nuclear localization signal (NLS) proteins and importin β1/karyopherin β1 (IPOB1 or KPNB1). These heterotrimeric complexes are responsible for transporting cargo from the cytoplasm to the nucleus. Mammals express six to seven KPNA subtypes, with specific isoforms exhibiting different expression levels in different cell types, including neuronal brain cells (1). KPNAs are vital in other cellular processes, including regulating stress granules and epigenetic processes (2, 3, 4). *Kpna1* (human importin α5; mouse importin α1) is expressed throughout the nervous system (1) and switching of *Kpna* subtype expression to *Kpna1* is essential in neuronal differentiation (5).

Owing to the high expression of KPNAs in the central nervous system (CNS) and peripheral nervous systems, and their various functions, investigating their dynamics in neurons is important. In humans, exome analysis of individuals with schizophrenia has revealed the presence of a rare *de novo* nonsense mutation in *Kpna1* related to disease pathogenesis (6, 7). Furthermore, *Kpna1*-KO mice demonstrate reduced anxiety-like behaviors (8), which are exacerbated in isolated social conditions or following subchronic administration of antipsychotic drugs, highlighting the significance of gene–environment interactions (9, 10). Single nucleotide polymorphisms in human *Kpna4* were found to reduce NF-κB activity and influence susceptibility to schizophrenia (11). In contrast, studies on *Kpna4* KO mice demonstrated psychiatric disorder–related behaviors associated with increased NF-κB activity (12). These findings emphasize the essential roles of various KPNA isoforms in neurons. However, the molecular mechanisms underlying psychiatric disorder onset remain unclear. Elucidating these mechanisms will provide novel insights into psychiatric conditions and will aid in developing novel treatment strategies.

Cargo transport along axons relies on molecular motors, such as cytoplasmic dynein 1 (hereafter referred to as dynein) and kinesins, which are essential for nerve development and function. Mutations in the genes encoding these essential transport machinery factors have been linked to neurological diseases (13); for instance, a mutation in the kinesin gene (*kif3b)* causes a schizophrenia-like phenotype (14). KPNAs are implicated in the retrograde transport of cargo from axons or dendritic spines to the cell body. In *Aplysia* neurons from the abdominal ganglion, proteins carrying an NLS signal can be retrogradely transported within the axon from the microinjected region to the nucleus, which is associated with injury signal transmission (15, 16, 17). Previous studies have demonstrated the involvement of KPNAs in the retrograde transport of NLS-harboring cargo from the injury site of peripheral nerves (18, 19, 20). While KPNAs may form a stable interaction with dynein, the local translation of IPOB1 within injured axons facilitates the formation of the dynein–importin–cargo complex (18, 19). Additionally, KPNA1 is anchored to the N-methyl-d-aspartate receptor (NMDAR) at synapses, the activation of which induces KPNA1 release, thereby enhancing the binding and transport of cargo from the synapse to the cell body (21). These importin-related retrograde transport processes are essential for the long-distance retrograde transmission of signals from remote injury sites or synapses to cell bodies.

Nevertheless, data regarding axonal importins and the exact dynamics of KPNA1 or IPOB1 within the axon remain limited. Previous studies have proposed the idea of rapid long-distance retrograde cargo transport within the axon (19, 22); however, the precise kinetics of this process remain to be demonstrated.

In this study, we hypothesize that the dynamics of KPNA and IPOB1 in axons are crucial in neuronal function. This study aims to address the following two key questions: “What are the precise dynamics of KPNA and IPOB1 within the axon?” and “How does the aberrant localization of mutated KPNA1 influence these dynamics and contribute to psychiatric conditions?”. Using peripheral neurons as a model, we observed and compared the dynamics of several axonal KPNAs and IPOB1 with those of dynein/dynactin complexes. Furthermore, we demonstrated that KPNA1 undergoes late endosome-dependent axonal transport and identified distinct importin dynamics between the cell body and axon. We also examined the dynamics of a mutated KPNA1 associated with the onset of schizophrenia. Our findings reveal the axonal dynamics of KPNAs and IPOB1, as well as the aberrant localization of the mutated KPNA1 protein. Our findings demonstrate that molecular importins in the axon exhibit a complex movement pattern that extends beyond retrograde signaling. This new insight into the novel motility of axonal importins provides valuable perspectives for understanding not only peripheral neurons but also central neurons, as well as the molecular underpinnings of neuropathies and psychiatric disorders.

## Results

### KPNA1 and importin-related proteins are expressed in peripheral neurons

**Figure 1:**
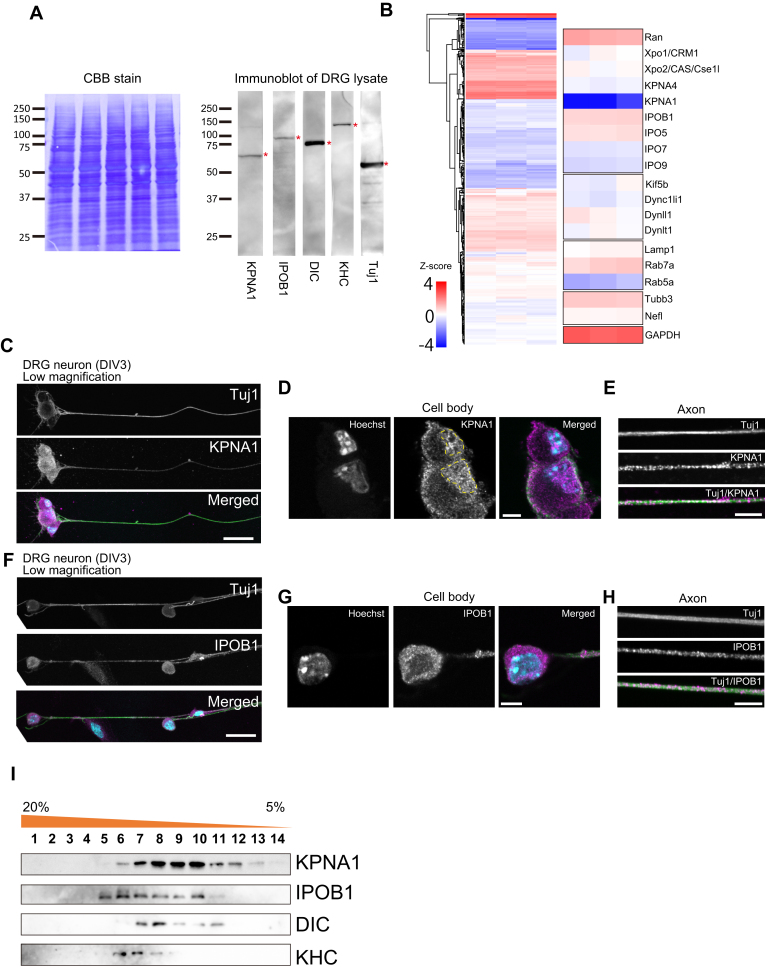
**Importin-related proteins are present in peripheral nerve axons**. *A*, immunoblots of the mouse dorsal root ganglia (DRG) lysate probed with anti-KPNA1, anti-IPOB1, anti-cytoplasmic dynein intermediate chain (DIC) and anti-Kinesin Heavy Chain (KHC), and the neuronal marker Tuj1 antibodies. Coomassie brilliant blue (CBB) staining on the *left* indicates the loading control of the sample. Asterisks (*red*) indicate the bands detected by the antibodies. *B*, heatmap of the z-score–transformed intensity values obtained through liquid chromatography–tandem mass spectrometry (LC-MS/MS) from the mouse DRG lysate. The label-free quantification (LFQ) intensity values for peptides were normalized, yielding z-scores from three replicates. *C*–*H*, immunofluorescence images of mouse DRG neurons at DIV3 probed with Tuj1 and KPNA1 (*C*–*E*) or IPOB1 (*F*–*H*) antibodies. (*C*, *F*): low magnification images (scale bar represents 25 μm). (*D*, *G*): magnified images of cell bodies (scale bar represents 5 μm). (*E*, *H*): magnified images of the axon (scale bar represents 5 μm). The *yellow* dashed lines indicate the position of the nucleus. *I*, sucrose density gradient centrifugation results. Following centrifugation, samples were fractionated and analyzed by immunoblotting with antibodies against KPNA1, IPOB1, DIC, and KHC.

To investigate the functionality of importins within neuronal axons, lysates were prepared from dorsal root ganglia (DRG) of mice, containing neuronal cell bodies including axons and associated glia cells and immunoblot analysis were performed (Fig. 1*A*). Anti-βIII Tubulin antibody/Tuj1 was used as a neuronal marker. Signals for KPNA1, IPOB1, and subunits of molecular motors, such as dynein and kinesin, were detected. Furthermore, we extracted the femoral nerve, which contains both motor and sensory axons, along with supporting structures, to analyze specific protein expression. Immunoblot analysis was conducted using antibodies against KPNA1, 2, 3, 4, 6, and IPOB1 with lysates from rat femoral nerves. KPNA1 exhibited a robust signal, whereas IPOB1 and KPNA3 displayed relatively weak signals (Fig. S1*A*). These results indicate the presence of KPNA1 in peripheral nerve lysates. Given the abundance within the nervous system and its involvement in neuronal function (1, 5, 19), our subsequent analyses focused on KPNA1. To assess importin-related proteins in peripheral nerve tissues, we performed liquid chromatography–tandem mass spectrometry (LC-MS/MS) on lysates from mouse DRGs. Several importin-related proteins were identified, including KPNA1, KPNA4, IPOB1, IPO5, IPO7, IPO9, CRM1/Exportin1, CAS/Exportin2, and the small GTPase Ran. These findings demonstrate the presence of nuclear transport factors in neurons, which are essential for shuttling between the nucleus and cytoplasm (Fig. 1*B* and Table S1), and confirm the localization of importin/exportin-related proteins within neurons. Notably, several members of the importin beta superfamily such as IPOB1 or IPO5 exhibited relatively higher intensities than importin alpha proteins (Fig. 1*B* and Table S1). These findings suggest the presence of diverse importin-related proteins within the peripheral nerves.

As the potential presence of material from adjacent glial cells in DRG or rat femoral nerve lysates could not be ruled out, we directly examined the presence of importins in axons. Primary cultured DRG neurons, that possess bifurcating axons but lack clear dendritic structures, were analyzed through immunofluorescence. KPNA1 and IPOB1 signals were detected within Tuj1-positive (Tuj1+) DRG neurons (Fig. 1, *C–H*). KPNA1 was localized in the axon of DRG neurons and cell bodies. In the cell bodies, KPNA1 was localized in the nucleus and weakly in the cytoplasm (Fig. 1*D*); however, in axons, it showed the punctate staining pattern (Fig. 1*E*). IPOB1 was localized in the cell bodies but not in the nucleus, with strong signals detected in the cytoplasm (Fig. 1*G*). In axons, IPOB1 showed the punctate staining pattern (Fig. 1*H*). We compared the expression of KPNA1 and IPOB1 between Tuj1+ neurons and other Tuj1-negative (Tuj1−) cells (Fig. S1, *B* and *C*). The expression levels of both KPNA1 and IPOB1 were significantly higher in Tuj1+ neurons than in Tuj1− cells, with KPNA1 showing higher expression in Tuj1+ neurons than in Tuj1− cells (Fig. S1*D*). Meanwhile, the difference in IPOB1 expression between Tuj1+ and Tuj1− cells was less pronounced (Fig. S1*D*), suggesting a more ubiquitous expression pattern for IPOB1. These results indicate the localization of KPNA1 and IPOB1 in neuronal axons. To assess the expression of other KPNAs, DRG neurons were probed with antibodies against KPNA1, 3, 4, and 6 (Fig. S1, *E* and *F*). KPNA1 exhibited a robust signal, consistent with the immunoblotting results (Figs. 1*A* and S1, *B* and *E*). KPNA3 also showed relatively high signals in immunofluorescence; however, immunoblotting results indicated that the expression of KPNA3 was lower than that of KPNA1 (Fig. S1, *A* and *F*). However, KPNA4 and KPNA6 signals were weaker than the KPNA1 signal, indicative of relatively low expression levels (Fig. S1, *A*, *E* and *F*). These results indicate that among KPNAs, KPNA1 shows the most robust signal not only in the cell body but also in axons.

To elucidate the characteristics of the KPNA1 and IPOB1 complexes, we fractionated rat femoral nerve lysates to analyze the axonal proteins through sucrose density gradient centrifugation and subjected the fractions to immunoblotting (Fig. 1*I*). The fractionation pattern of KPNA1 was similar to that of the cytoplasmic dynein intermediate chains (DIC). Conversely, the elution peak of KPNA1 differed from that of kinesin heavy chain (KHC). IPOB1 exhibited a distinct fractionation pattern compared with KPNA1, indicating separate KPNA1 and IPOB1 complexes in the axon. While the elution peak of IPOB1 was distinct from the peak of DIC1, it could be similar to that of KHC.

To validate the interaction of KPNA1 with dynein and IPOB1, we performed immunoprecipitation using anti-KPNA1 and anti-DIC antibodies on whole mouse brain and rat femoral nerve lysates. Although DIC efficiently precipitated from brain lysates using anti-DIC antibodies, it did not coprecipitate with anti-KPNA1. Similarly, anti-DIC antibodies did not efficiently coprecipitate KPNA1 protein, with only a small amount of KPNA1 (Fig. S2*A*). These results suggest that the interaction between KPNA1 and DIC may not necessarily be sufficiently stable to precipitate their binding partners. In rat femoral nerve lysates, KPNA1 and IPOB1 were individually immunoprecipitated by their respective antibodies. However, KPNA1-IP did not coprecipitate IPOB1, and IPOB1-IP did not coprecipitate KPNA1, indicating that the interaction between KPNA1 and IPOB1 could not be confirmed using immunoprecipitation (Fig. S2, *A* and *B*). These results do not support the interaction between KPNA1 and IPOB1 within the axons at least in peripheral neurons in a noninjured and stable state.

### Distinct axonal localization of KPNAs and IPOB1

**Figure 2:**
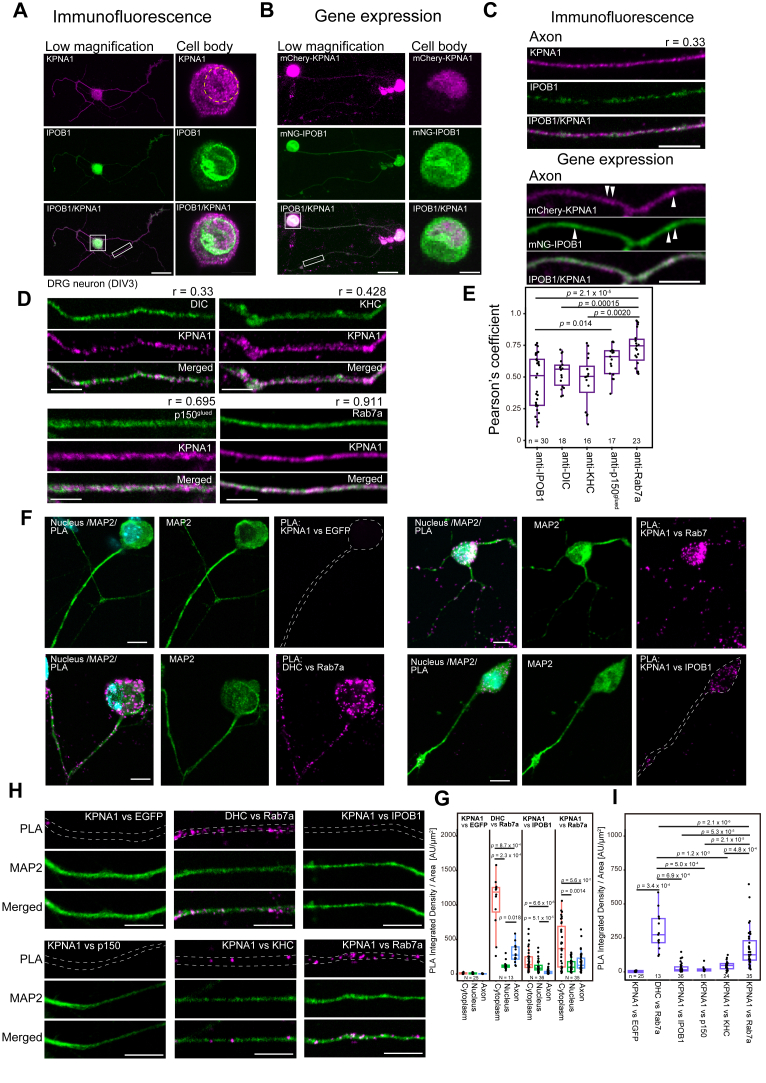
**Neuronal KPNA1 localizes in axons with a punctate pattern correlated with endosomes**. *A*, immunofluorescence images of mouse DRG neurons probed with anti-KPNA1 and anti-IPOB1 antibodies. A low magnification image (scale bar represents 25 μm) and a magnified image of the cell body (scale bar represents 5 μm) are shown. For the low-magnification image, a maximum projection of the Z-stack is shown. For the magnified images of the cell body and the axon, a single plane from the Z-stack is shown. The magnified cell body region and the axons are each indicated by a *white* box. *B*, DRG neuron transfected with mCherry-KPNA1 and mNG-IPOB1. A low magnification image (scale bar represents 25 μm) and a magnified image of the cell body (scale bar represents 5 μm) are shown. The cell body signals in the low-magnification images of (*A*) and (*B*) are saturated to highlight the axonal signals. *C*, magnified images of axons from immunofluorescence (above) or gene expression (below). The Pearson’s correlation coefficients from the correlation analysis for immunofluorescence images are indicated in the *right* corner of the image (*top*). Scale bar represents 5 μm. *D*, co-immunofluorescence images of mouse DRG axons probed with DIC, KHC, p150^glued^, and Rab7a antibodies, all co-stained with anti-KPNA1. Scale bar represents 5 μm. The Pearson’s correlation coefficients from the correlation analysis for immunofluorescence images are indicated in the *right* corner of the image (*top*). Note that each value is a representative value from the shown small region of images in (*D*) and is not identical to the values analyzed in (*E*). *E*, comparison of Pearson’s correlation coefficients for colocalization. Values from axonal region of co-immunofluorescence images of KPNA1 and the indicated antibodies are shown. The *p*-values, calculated using Welch's *t* test with Holm's correction for multiple comparisons, are indicated above each bar. The number of analyzed cells is shown at the bottom of each column. Data are derived from two independent experimental replicates. *F*, proximity ligation assay (PLA) images of DRG neurons. PLA signals, anti-MAP2 (microtubule-associated protein 2, neuron marker), and merged images are shown. Combinations of antibodies are indicated above. Scale bar represents 10 μm. Cell bodies and axons are outlined in the PLA images. Scale bar represents 10 μm. *G*, quantification of integrated intensity/area (A.U./μm^2^) of PLA dots for combination of KPNA1 with EGFP, IPOB1, and Rab7a in DRG neurons. PLA signal intensities in the cytoplasm, nucleus, and axons were compared. The *p*-values, calculated using Welch's *t* test with Holm's correction for multiple comparisons, are indicated above. The number of analyzed cells is shown at the *bottom* of each column. Data are derived from two independent experimental replicates. *H*, magnified images of PLA experiments with combination of KPNA1 and EGFP, DHC and Rab7a, KPNA1 and IPOB1, KPNA1 and p150^glued^, KPNA1 and KHC, KPNA1, and Rab7a antibodies indicated. The region of axons is outlined with *white dashed* lines. Scale bar represents 10 μm. *I*, quantification of integrated intensity/area (A.U./μm^2^) of PLA dots in axonal regions. A combination of antibodies is shown at the *bottom*. The number of analyzed cells is indicated at the *bottom* of each column. The *p*-values, calculated using Welch's *t* test with Holm's correction for multiple comparisons, are indicated above each bar. The number of analyzed cells is indicated at the *bottom* of each column. Data are derived from two independent experimental replicates. DHC, dynein heavy chain.

To assess the colocalization of KPNA1 and IPOB1 in neurons, immunostaining was performed using their respective antibodies simultaneously on DRG neurons (Fig. 2*A*). In the cell body, KPNA1 was predominantly localized in the nucleus, with relatively weak localization in the cytoplasm (Fig. 2*A*, top). Meanwhile, IPOB1 showed localization in the cytoplasm and clear localization at the nuclear membrane; however, little localization was observed within the nucleus (Fig. 2*A*, middle). In axons, KPNA1 showed a robust signal (Fig. 2, *A* and *C*, top of upper panels). Although IPOB1 showed a relatively lower signal than KPNA1, a certain degree of localization was observed in axons (Fig. 2, *A* and *C*, middle of upper panels). Their colocalization was rarely observed along the axon and their staining patterns exhibited dissimilar features (Fig. 2*C*, bottom of upper panels). To investigate the localization of KPNA1 and IPOB1 in live cells of DRG neurons, we analyzed the localization of the fusion proteins in neurons transfected with mNeonGreen (mNG)-IPOB1 and mCherry-mKPNA1 (Fig. 2*B*). Live-cell imaging of neurons showed that mCherry-KPNA1 accumulated in the nucleus and was localized in the cell body and axons, while mNG-IPOB1 was localized in the nuclear membrane, in the cell body, and the axon of neurons, showing a similar localization pattern with internal KPNA1 or IPOB1 detected with immunofluorescence (Fig. 2, *A* and *C*, upper panels). The axonal localization patterns of both mNG-IPOB1 and mCherry-KPNA1 appeared punctate though less clear than that of immunostaining, while the colocalization of KPNA1 and IPOB1 was not detected in the live imaging of both proteins (Fig. 2, *B* and *C*, lower panels). These findings confirmed that interaction between KPNA1 and IPOB1 was limited or low despite their overexpression and/or abundance in the axonal region. These results indicate that KPNA1 and IPOB1 are not completely colocalized in the axons of DRG neurons even in gene-transduced DRG neurons in which sufficient amount of IPOB1 exists in the axons, corroborating the outcomes of our biochemical investigations (Figs. 1*I* and S2, *A* and *B*).

To investigate the relative localization and potential association between KPNA1 and axonal motile complexes, we performed immunostaining using anti-KPNA1 and several related proteins (Fig. 2*D*) and analyzed the degree of colocalization using Pearson’s correlation coefficient (Fig. 2*E*). Among the numerous punctate signals of KPNA1, neither DIC nor KHC exhibited clear or complete colocalization, showing only limited overlap with DIC or KHC (Fig. 2*D*). Pearson’s correlation coefficients, which were approximately 0.3 to 0.5, indicated a weak correlation between KPNA1 and the signals of dynein or kinesin. In contrast, the antibody against a subunit of dynactin, p150^glued^, demonstrated a relatively high degree of colocalization, as supported by Pearson’s correlation coefficient values (Fig. 2, *D* and *E*). Additionally, we analyzed endosomes, which represent motility complexes in axons using various antibodies against an endosome marker. Signals for a late endosome marker, Rab7a, were detected. Rab7a displayed similar staining patterns with KPNA1 within axons (Fig. 2*D*). Statistical comparisons revealed that Pearson’s coefficients for Rab7a were significantly higher than those for IPOB, DIC, and KHC (Fig. 2*E*). Based on these results, KPNA1 and dynein or kinesin exhibit related localization rather than directly interacting with each other, and KPNA1 motility along the axon may depend on their localization within axonal endosomes.

To analyze the direct interaction between KPNA1 and the related proteins, we performed a proximity ligation assay (PLA) using anti-KPNA1, IPOB1, and various other antibodies. PLA against NIH3T3 cultured cells with KPNA1 and IPOB1 antibodies revealed the presence of PLA dots exclusively in the cytoplasm, with very few in the nucleus (Fig. S2*C* and Video S1). The quantification of integrated intensity values of dots per area demonstrated significantly higher intensities in the cytoplasm than in the nucleus (Fig. S2*D*). These results indicate that the direct interaction between KPNA1 and IPOB1 was restricted to the cytoplasm and suggest no direct interaction in the nucleus. Furthermore, PLA was performed against DRG neurons. Negative control experiments with anti-KPNA1 and anti-EGFP antibodies showed no PLA dots in the cytoplasm, nucleus, or axon (Fig. 2*F*). The quantification of integrated intensity values of PLA dots indicated that the number of dots in PLA with KPNA1 and EGFP was minimal in the cytoplasm, nucleus, and axons (Fig. 2*G*). In contrast, the combination of dynein heavy chain (DHC) and Rab7a, which were expected to interact in the axon *via* Rab-interacting lysosomal protein and p150^glued^ (23), showed many PLA dots both in the cytoplasm and axons (Fig. 2*F*) and a significantly high number of intensity values per area in cytoplasm and axons but not in the nucleus (Fig. 2*G*). PLA with KPNA1 and IPOB1 antibodies showed multiple PLA dots in the cytoplasm; however, PLA dots were rarely observed in axons or in the nucleus (Fig. 2*F*). This, as indicated in the related Z-series video (Video S2), was not attributed to the focus position of the microscope. Quantification further showed that the intensity of PLA dots in the axon of DRG neurons was significantly lower than that in the cytoplasm (Fig. 2*G*). We analyzed the KPNA1 and various proteins with PLA. In PLA with KPNA1 and p150^glued^ or KPNA1 and KHC, the PLA dots were rarely observed in the axon (Fig. 2*H*). Quantification showed that the values in the axon were not significantly different from KPNA1 versus IPOB1 (Fig. 2*I*). Meanwhile, a large number of PLA dots were observed in the PLA with the combinations of KPNA1 and Rab7a (Fig. 2*H*). Quantification showed that values for KPNA1 and Rab7a were significantly higher than those for KPNA1 and EGFP or KPNA1 and IPOB1 and comparable to the values for DHC and Rab7a (Fig. 2*I*). These results suggest that KPNA1 localizes in axons through mechanisms other than direct interaction with dynein, and the localization of KPNA1 in axon may be dependent on the endosomes. Additionally, PLA was performed to confirm the co-existence of IPOB1 and molecular motors. PLA dots from the combination of IPOB1 and DHC, or IPOB1 and KHC were rarely observed in the axonal regions of DRG neurons (Fig. S2*E*). Quantification showed that PLA signals from these combinations were low and not significantly different from negative control (KPNA1 and EGFP) (Fig. S2*F*). These results suggest that IPOB1 does not directly interact with molecular motors in the axons of neurons.

### Stable but fluctuating dynamics with bidirectional switching of axonal KPNAs and IPOB1

**Figure 3:**
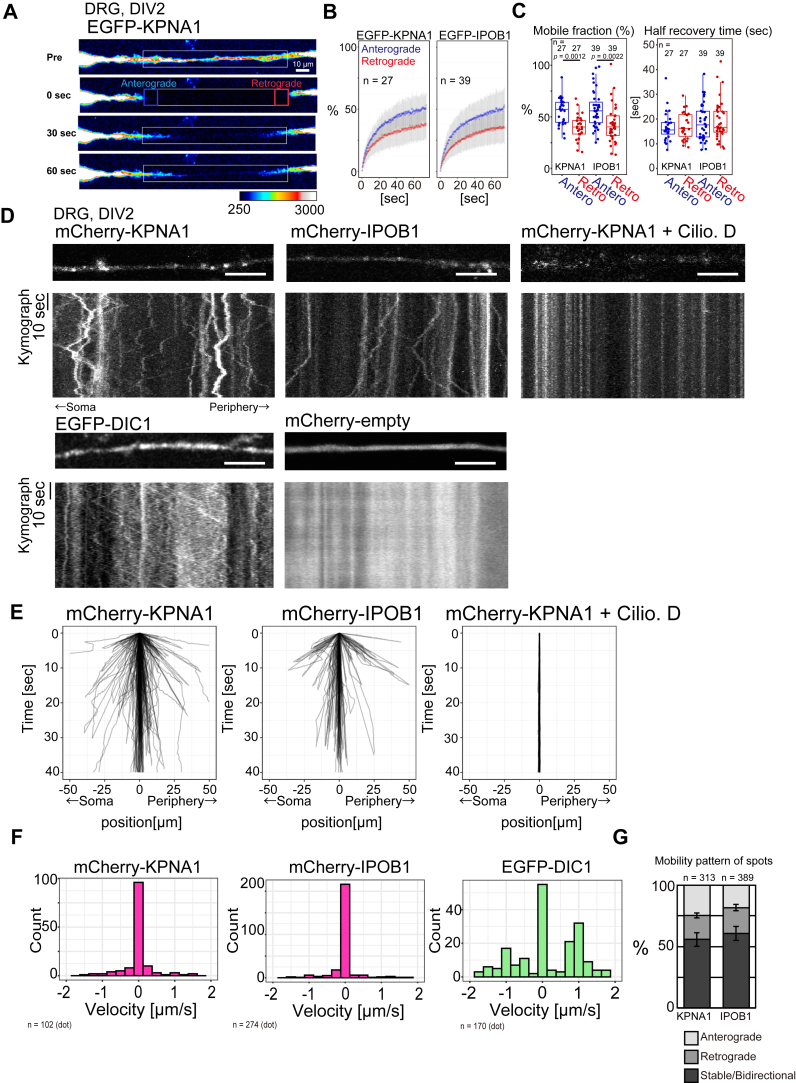
**Axonal KPNA1 and IPOB1 actively migrate in the axon**. *A*, representative images of fluorescence recovery after photobleaching (FRAP) analysis of dorsal root ganglia (DRG) neurons transfected with EGFP-KPNA1. The area under the *white* rectangular region was bleached and fluorescence recovery was monitored in the anterograde (*blue* box) and retrograde (*red* box) directions. *B*, graphs showing the time course of relative fluorescent recovery after bleaching of the signal from DRG neurons transfected with EGFP-KPNA1 (*left*) or EGFP-IPOB1. Pre-bleach level is normalized to 100%. Data are shown as mean ± SD. Measurements were obtained from 27 and 39 cells and experiments were independently performed three times. *C*, comparison of parameters obtained from FRAP analysis, including the mobile fraction (*left*) or half-time for fluorescence recovery (*right*). The *p*-values, calculated using Wilcoxon's rank sum test with Holm's correction for multiple comparisons, are shown. Measurements were obtained from 27 or 39 cells and experiments were independently performed three times. *D*, representative kymographs of the axonal region transfected with mCherry-KPNA1, mCherry-IPOB1, EGFP-DIC1, and mCherry-empty plasmids. Effects of 25 μM Ciliobrevin D. (Cilio. D.) was tested. *E*, trajectories of fluorescents spots of mCherry-KPNA1, mCherry-IPOB1, and mCherry-KPNA1 with 25 μM Ciliobrevin D in DRG neuron axons. Trace numbers are 169, 81, and 84, obtained from 19, 8, and 12 cells, respectively, across two or three independent experiments. *F*, histogram of velocities from mCherry-KPNA1, mCherry-IPOB1, and EGFP-DIC1 fluorescent spots in DRG neuron axons. Spot numbers for histograms are 102, 274, and 170, obtained from 21, 21, and 13 cells, respectively. Data are derived from three independent experimental replicates. *G*, quantification of the mobility patterns of mCherry-KPNA1 and mCherry-IPOB1 fluorescent spots. Fluorescent spots migrating with the mean velocity (60 s) more than + 0.03 μm/s or −0.03 μm/s were classified as anterograde or retrograde, respectively; others were classified as stable or bidirectional movements. Experiments were repeated three times, and mean percentages are shown. Error bars indicate the SEM. The measurements are from 313 or 389 fluorescent spots across 31 or 39 cells, respectively.

To investigate KPNA1 and IPOB1 dynamics within the axons, we imaged DRG neurons transfected with EGFP or mCherry fusion genes. To determine whether KPNA1 and IPOB1 exhibit mobility along the axons, we utilized fluorescence recovery after photobleaching (FRAP; Fig. 3*A* and Video S3) (24, 25). A long rectangular region along the axon underwent bleaching, and fluorescence recovery was monitored to analyze anterograde or retrograde protein movement. Anterograde and retrograde directions were distinguished based on the position of the cell body or nerve terminal. Anterograde and retrograde fluorescence recovery were observable, signifying the high mobility of the fusion proteins (Fig. 3, *A*–*C*). Both EGFP- or mCherry-fused KPNA1 and EGFP-IPOB1 were examined, and parameters, such as the half-time of recovery (t_1/2_) and the percentage of mobile fractions, were compared (Fig. 3, *B* and *C*, and Fig. S3, *A* and *B*). KPNA1 and IPOB1 displayed a higher percentage of mobile fractions in the anterograde than in the retrograde directions (Fig. 3, *B* and *C*, and Fig. S3, *A* and *B*). No significant disparities in t_1/2_ values were observed between the anterograde and retrograde directions. These findings suggest that axonal KPNA1 and IPOB1 exhibit pronounced mobility in the retrograde and anterograde directions. The high percentage of the mobile fraction in anterograde fluorescence recovery could be attributed to the transport activities of anterograde kinesin movement. Although the diffusion proteins from the cell body might have additive effects in these directions, we have previously indicated that the impact of diffusion was minimal (24). Furthermore, FRAP experiments using KPNA3, 4, and 6 yielded results consistent with those for KPNA1, implying comparable mobility patterns among the KPNAs in the axonal region (Fig. S3, *A* and *B*). These results suggest that axonal KPNAs are mobile in both the retrograde and anterograde directions.

To assess the mobility of punctate KPNAs/IPOB1 within axons, we examined their motility through kymography analysis of mCherry-KPNAs and IPOB1 and their trajectories (Fig. 3, *D* and *E*). DRG neurons expressing mCherry-KPNA1 and mCherry-IPOB1 exhibited mobile punctate patterns along the axons, consistent with the immunofluorescence findings (Fig. 3*D*, Video S4). Neurons expressing mCherry alone *via* an empty expression vector displayed a distinctive pattern of protein localization and mobility in the axons (Fig. 3*D* and Video S5, top). These findings suggest that the observed localization patterns were not artifacts from the fusion with fluorescent proteins. The kymographs indicated that numerous spots remained stable in their mobility and displayed bidirectional fluctuations in position with switching in their moving directions (Fig. 3, *D* and *E*). Introducing Ciliobrevin D, a dynein inhibitor, effectively halted all axonal motility (Fig. 3, *D* and *E,* and Video S5, middle). This bidirectional inhibition of axonal transport is consistent with previous findings showing that the both anterograde and retrograde motility are inhibited by the Ciliobrevin D treatment (26), emphasizing the dependence of bidirectional KPNA1 mobility on molecular motor activity. These findings suggest that axonal KPNA1 bidirectional motility with frequent switching in the directions relies on the activity of molecular motors such as kinesin, not limited to the binding to dynein, *via* direct or indirect interactions.

Observation of EGFP-DIC1 revealed diverse motility patterns, with several anterograde- or retrograde-directed movements appearing as diagonal lines, indicative of motion with relatively uniform velocity (Fig. 3*D*, Video S5, bottom). In contrast, mCherry-KPNA1 and mCherry-IPOB1 kymographs with uniform velocities were rarely observed compared with those of EGFP-DIC1. Analysis of their trajectory velocities and comparison with those of EGFP-DIC1, mCherry-KPNA1, and mCherry-IPOB1 revealed anterograde (denoted by positive values) and retrograde motilities (denoted by negative values) (Fig. 3, *E* and *F*). The histogram of average trajectory velocities of EGFP-DIC1 displayed a trimodal pattern, representing anterograde, retrograde, and proteins with relatively stable or bidirectional motility. Typical velocities for anterograde or retrograde movement of EGFP-DIC1 were approximately 1 and −1 μm/s, respectively, consistent with the speed of kinesin or dynein in the axon. In contrast, the velocity histograms of KPNA1 and IPOB1 displayed a monomodal distribution, suggesting that many axonal KPNA1 particles showed relatively stable motion.

Low net average velocities could be attributed to the averaging of bidirectional motility (Fig. 3*F*). We compared the highest and lowest velocities to assess instantaneous velocity *via* the analysis of maximal or minimal values of mCherry-KPNA1/IPOB1 velocities (Fig. S4). Minimal values represent the fastest motility of spots toward retrograde directions, and maximal values represent the fastest motility toward anterograde directions from their trajectories. Several fluorescent spots of KPNA1 or IPOB1 actively transiently migrated toward anterograde or retrograde directions (Fig. S4), though IPOB1 was more static than KPNA1 from the histogram (Fig. S4*B*). Those results suggest that the same KPNA1 fluorescent spots exhibited transiently fast anterograde- or retrograde-directed migration while switching directions. This behavior signifies heterogeneous motility among KPNA1 or IPOB1 fluorescent spots, including stable motility and instantaneous active migration with fluctuations (Fig. 3, *E* and *F*, and Fig. S4). The motility patterns of both KPNA1 and IPOB1 were categorized as anterograde, retrograde, or stable/bidirectional. Over 50% of the spots for both KPNA1 and IPOB1 demonstrated stable/bidirectional motility with switching directions suggesting that the migration of axonal KPNA1 was slow and stable (Fig. 3*G*). Additionally, kymography analysis of KPNA3, 4, and 6 yielded consistent results with those of KPNA1, demonstrating relatively stable motility patterns (Fig. S3*C*, *D*). Collectively, these results suggest that axonal importins are mobile within axons and are characterized by heterogeneous motility patterns encompassing numerous stable/bidirectional spots with net slow migration.

### KPNA1 comigrates with axonal endosomes/lysosomes

**Figure 4:**
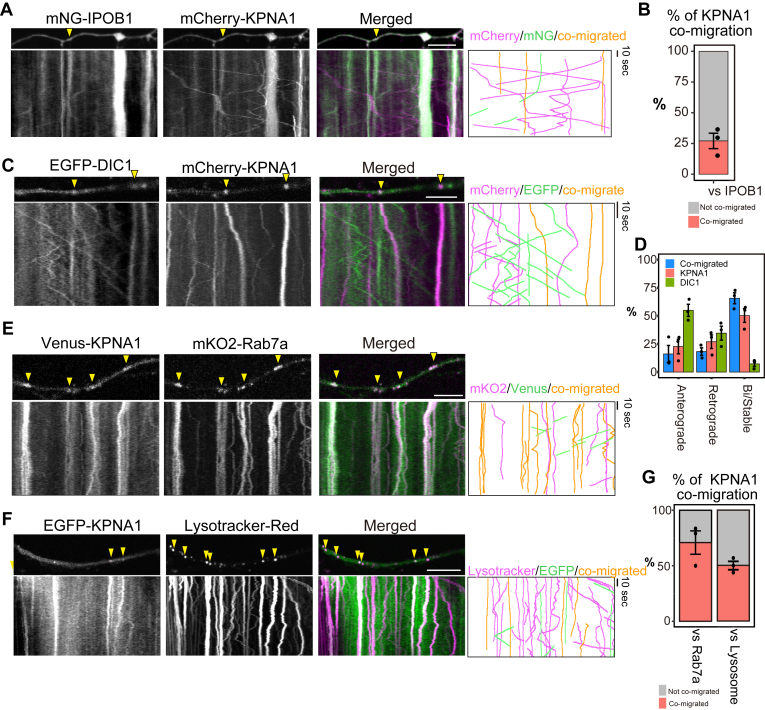
**KPNA1 does not interact with IPOB1 or cytoplasmic dynein directly**. *A*, *C*, *E*, and *F*, kymograph of dual-color live imaging and extracted trajectories (*right*). *Green* trajectories represent non-co-migrated mNG, EGFP, or Venus fusion proteins. *Magenta* trajectories represent non-co-migrated mCherry, mKO2, or LysoTracker. *Orange* trajectories indicate comigration of signals from both wavelengths. Scale bar represents 10 μm. *A*, axon of DRG neurons transfected with EGFP-KPNA1 and mNG-IPOB1. Kymographs of fluorescent spots are shown at the *bottom*. Comigrating traces are indicated by arrowheads. *B*, quantification of the motility pattern of comigrating (*magenta*) or independently migrating fluorescent spots (*gray*). Mean percentages from three independent experiments are shown. The measurements are derived from 314 fluorescent spots of mCherry-KPNA1, analyzed across 30 cells. Data are collected from three independent experimental replicates. *C*, axon of DRG neurons transfected with EGFP-DIC1 and mCherry-KPNA1. Kymographs of fluorescent spots are shown at the *bottom*. Comigrating traces are indicated by arrowheads (*yellow*). *D*, quantification of the motility pattern of comigrating (*blue*) or independently migrating fluorescent spots. *Orange* and *green* indicate KPNA1 and DIC1, respectively. Mean percentages from three independent experimental replicates are shown. The measurements are derived from 351 fluorescent spots analyzed across 18 cells. *E* and *F*, axon of DRG neurons transfected with (*E*) Venus-KPNA1 and mKO2 Rab7a or (F) EGFP-KPNA1 and treated with LysoTracker-Red. Kymographs of fluorescent spots are shown at the *bottom*. Comigrating traces are indicated by arrowheads (*yellow*). *G*, quantification of the mobility patterns of comigrating (orange) and independently migrating fluorescent spots (*gray*) of KPNA1. Mean percentages from three independent experiments are shown. The measurements are derived from 19 cells and 130 fluorescent spots (*left*) and 16 cells and 88 fluorescent spots (*right*).

To examine the relationship between KPNAs and IPOB1 in axons, dual live imaging was conducted in DRG neurons expressing mNG-IPOB1 and mCherry-KPNA1. Comigration analysis showed that the number of mCherry-KPNA1 that comigrated with mNG-IPOB1 was limited (Fig. 4*A* and Video S6). Quantitative analysis revealed that the proportion of kymographs with colocalized tracks in two colors was approximately 25% for mCherry-KPNA1 and mNG-IPOB1 (Fig. 4*B*). Notably, in our experimental setup, both KPNA1 and IPOB1 were abundant in the axons. This implies that despite their abundance, not all axonal KPNA1 and IPOB1 primarily migrate as part of the same protein complexes.

To analyze the relationship between the motilities of axonal KPNAs and molecular motor complexes, dual live imaging experiments were conducted using dynein or dynactin-related fusion proteins, such as EGFP-DIC1 or EGFP-p150^glued^, and their comigration was analyzed. EGFP-DIC1 and EGFP-p150^glued^ exhibited mixed motility patterns, including motions with uniform velocities and low signal intensities, as well as stable or bidirectional slow transport with higher signal intensities with directional switching (Figs. 4*C*, S4*C*, Video S7, and S8). In contrast, mCherry-KPNA1 kymographs revealed that most of its motility was stable/bidirectional (Figs. 4*C* and S4*C*). Comigration analysis of EGFP-DIC1 and EGFP-p150^glued^ with mCherry-mKPNA1 revealed that many dynein-related proteins did not show clear comigration with mCherry-KPNA1, while a limited number of KPNA1 spots exhibited a comigrated pattern (Figs. 4*C* and S4*C*). Quantitative analysis of motility patterns indicated that over 50% of the fluorescence spots, wherein EGFP-DIC1 or EGFP-p150^glued^ comigrated with mCherry-KPNA1, underwent bidirectional and stable transport along the axon, exhibiting slow or stable net migration (Figs. 4*D* and S4*D*). These findings suggest that some axonal KPNA1 can comigrate with dynein and dynactin complexes and be subject to slow transportation by molecular motors. Similar patterns were observed when analyzing the motility of mCherry-KPNA3, 4, and 6 using dual live imaging with EGFP-DIC1 or EGFP-p150^glued^ (Fig. S5). Collectively, these results indicate that axonal importins exhibit mobility within axons and are characterized by heterogeneous motility patterns; however, most of them were stable in steady state noninjured axons. These patterns encompass numerous stable or bidirectional movements with net slow migration, interspersed with prolonged stationary periods.

To explore the potential relationship between axonal KPNAs and other axonal structures, we focused on axonal endosomes/lysosomes, which typically exhibits mixed patterns of stable and bidirectional motility. Some of KPNA1 fluorescent spots showed relatively high fluorescence intensity and stable movement; however, they sometimes showed instantaneous mobility toward anterograde or retrograde directions (Fig. 3*D*). These features correspond well with those of the axonal endosomes or lysosomes (27). To determine whether if KPNA1 comigrates with endosomes/lysosomes along the axon, live-cell imaging of Venus/EGFP-KPNA1 was conducted using mKusabira-Orange 2 (mKO2)-Rab7a or LysoTracker-Red. Kymographs demonstrated relatively similar motility patterns between KPNA1 and Rab7a or lysosome, supporting their comigration (Fig. 4, *E* and *F*, and Video S9). Quantitative analysis indicated that > 50% of the fluorescent spots exhibited comigration with Rab7a; a slightly low percentage was indicated by LysoTracker-Red (Fig. 4*G*). These results suggest that some of KPNA1 are transported along with vesicular cargos, supporting the notion that axonal importins encompass both vesicular and nonvesicular complexes.

### Effects of schizophrenia-related mutation on KPNA1 localization

**Figure 5:**
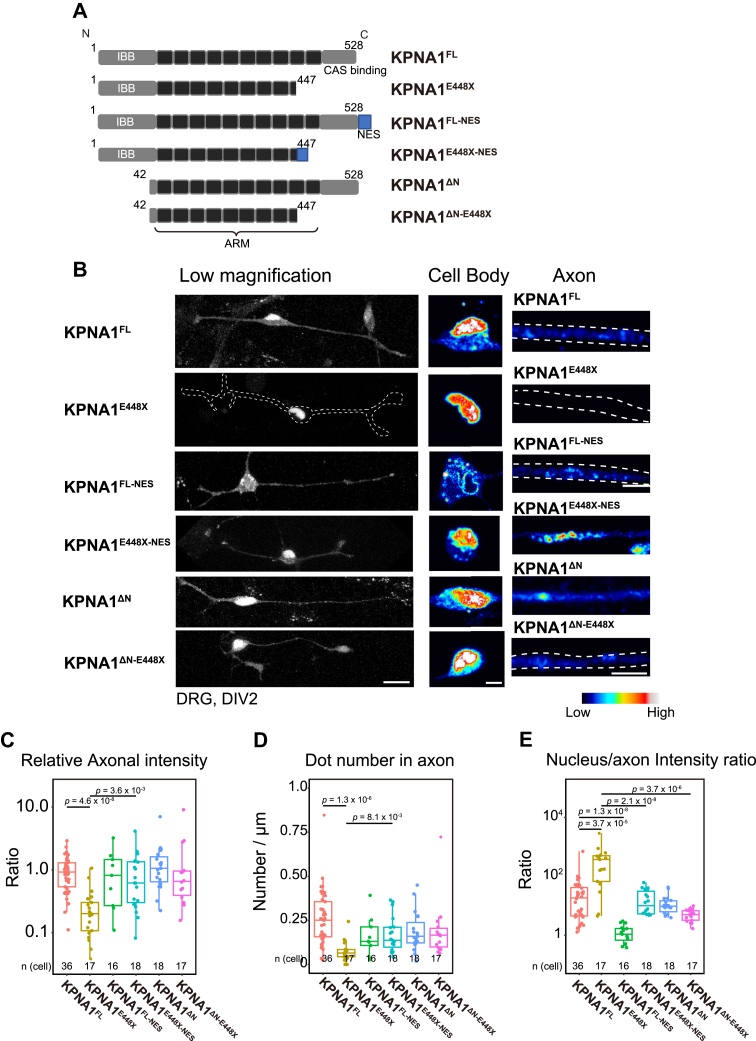
**Schizophrenia-related KPNA1 mutation caused abnormal nuclear accumulation and localization**. *A*, schematic representation of the KPNA1 domain structure of full-length KPNA1 (KPNA1^FL^), E448X mutation (KPNA1^E448X^), full-length KPNA1 conjugated with nuclear export sequence (NES) (KPNA1^FL-NES^), E448X mutation protein conjugated with NES sequence (KPNA1^E448X-NES^), KPNA1 with an N terminus deletion (KPNA1^ΔN^), and KPNA1 with both an N terminus deletion and the E448X mutation (KPNA1^ΔN-E448X^). The numbers above each bar represent the positions of the corresponding amino acid residues. *B*, dorsal root ganglia (DRG) neurons transfected with various KPNA1. Low magnification images (*left*, maximal projection of XYZ stack) and magnified images of the cell body (*middle*, single plane of the stack) and axon (*right*, single plane of the stack) are shown. The shape of the cell (*left*) or axon (*right*) is indicated by a *white dotted* line. Scale bar represents 20 μm (low magnification) and 5 μm (magnification for cell body and axon). *C*, comparison of the relative intensity values in the axons of DRG neurons transfected with various mCherry-KPNA1 constructs. Data were normalized to the mean value of KPNA1^FL^. The number of analyzed cells is indicated at the *bottom* of each column, with data collected from three independent experimental replicates. The *p*-values are indicated at the *top* of each column. Statistical analysis was performed using Wilcoxon's rank sum test with Holm's correction for multiple comparisons. *D*, comparison of the mean dot number per 1 μm of the axonal region in DRG neurons transfected with various mCherry-KPNA1. Cell numbers are indicated at the *bottom* of each column, with data collected from three independent experimental replicates. The *p*-values are shown at the top of each column. Statistical analysis was performed using Wilcoxon's rank sum test with Holm's correction for multiple comparisons. *E*, comparison of intensity ratios between nucleus and axon in DRG neurons transfected with various mCherry-KPNA1 constructs. The number of analyzed cells is indicated at the *bottom* of each column, with data collected from three independent experimental replicates. The *p*-values are indicated at the *top* of each column. Statistical analysis was performed using Wilcoxon's rank sum test with Holm's correction for multiple comparisons. ARM, armadillo repeat; IBB, importin β–binding motif; NES, nuclear export sequence.

Exosome screening of patients with schizophrenia identified a rare *de novo* nonsense mutation (E448X) in KPNA1 (6, 7). Using our peripheral neuron system with DRG neurons, we examined the localization of mCherry-KPNA1^E448X^ in DRG neurons to gain comprehensive insights into the effects of a mutation in *Kpna1* gene and the localization and motility of the KPNA1 protein in axons (Fig. 5, *A* and *B*). Unlike full-length KPNA1 (KPNA1^FL^), KPNA1^E448X^ accumulated exclusively in the nucleus, with no or very weak fluorescence observed in the cytoplasm of the cell body or at the axonal region of DRG neurons (Fig. 5*B*). Quantitative analysis of relative intensity values of axonal KPNA1 and the number of fluorescent spots revealed significant reductions in mCherry-KPNA1^E448X^ in the axonal region (Fig. 5, *C* and *D*). These findings indicate the loss of axonal localization of KPNA1 in mCherry-KPNA1^E448X^. When comparing relative nuclear and axonal KPNA1 intensities, KPNA1^E448X^ exhibited an elevated nuclear/axonal fluorescence intensity ratio, which is significantly higher than the value of the KPNA1^FL^ protein, confirming the significant nuclear accumulation of mutated KPNA1 and the loss of axonal KPNA1 (Fig. 5*E*). This abnormal protein accumulation in the nucleus likely results from the loss of KPNA1 export from the nucleus, considering that the C-terminus of KPNA1 facilitates CAS exportin binding (28, 29).

To assess the effects of the E448X mutation on the KPNA1 protein in the axonal region and restore the mutated axonal localization of the protein, a chimera gene was generated by linking the *Kpna1* gene to the nuclear export sequence (NES) of the protein kinase inhibitor to determine whether the localization of KPNA1^E448X^ could be rescued by this addition (Fig. 5*A*) (30). KPNA1 with NES connected to the C-terminus of full-length KPNA1 (KPNA1^FL-NES^) and E448X (KPNA1^E448X-NES^) localized in the axon (Fig. 5, *B* and *E*). Their relative intensities and spot numbers did not significantly differ from those of KPNA1^FL^ (Fig. 5, *C* and *D*). In the cell body, KPNA1^FL-NES^ showed loss of protein from the nucleus (Fig. 5, *B* and *E*), suggesting that the activity of both CAS and CRM1 exportins on KPNA1 excluded the fusion protein from the nucleus. The addition of NES to E448X cells significantly increased axonal intensity and spot density, and the nucleus-to-axon ratio was restored to the level of KPNA1^FL^, implying that NES rescued abnormal KPNA1^E448X^ localization (Fig. 5, *B*–*E*). Furthermore, a protein with a 41-amino acid N-terminal deletion was generated, resulting in the loss of most of the importin ß binding (IBB) domain (KPNA1^ΔN^) and ΔN-E448X protein (KPNA1^ΔN-E448X^) (Fig. 5*A*). The relative axonal KPNA1 intensities or spot numbers did not significantly differ from those of the full-length protein in either KPNA1^ΔN^ or KPNA1^ΔN-E448X^ (Fig. 5, *B*–*D*). However, the nucleus-to-axon ratio was significantly lower in KPNA1^ΔN-E448X^ than in KPNA1^E448X^ (Fig. 5*E*), indicating that the binding of IPOB1 to the IBB domain of KPNA1 is necessary for abnormal accumulation of KPNA1^E448X^.

### Restoration of mutant KPNA1 motility *via* NES fusion

**Figure 6:**
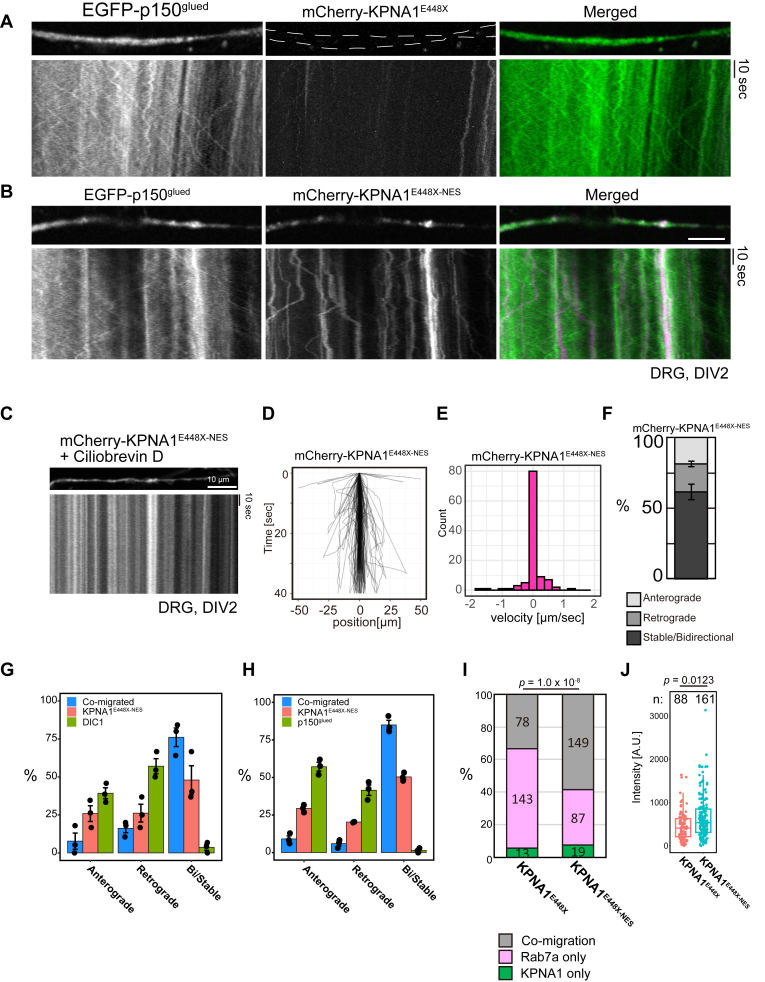
**Addition of NES rescues motility impairment caused by the schizophrenia-related KPNA1 mutation**. *A*, axon of dorsal root ganglia (DRG) neurons transfected with EGFP-p150^glued^ and mCherry-KPNA1^E448X^ (*top*) and kymographs of fluorescent spots (*bottom*). *B*, axon of DRG neurons transfected with EGFP-p150^glued^ and mCherry-KPNA1^E448X-NES^ (*top*) and corresponding kymographs of fluorescent spots (*bottom*). *C*, axon of DRG neurons transfected with mCherry-KPNA1^E448X-NES^ treated with Ciliobrevin D. *D*, trajectories of fluorescents spots of mCherry-KPNA1^E448X-NES^. Measurements are derived from 100 fluorescent spots analyzed across 17 cells, with the experiment repeated independently three times. *E*, histogram of velocities from mCherry-KPNA1^E448X-NES^ fluorescent spots in the axon of DRG neurons. Measurements are derived from 113 fluorescent spots analyzed across 21 cells, from three independent experimental replicates. *F*, quantification of mCherry-KPNA1^E448X-NES^. The experiments were repeated three times and mean percentages are shown. Error bars indicate the SEM. Measurements are from 113 fluorescent spots from 21 cells. *G* and *H*, quantification of the motility pattern of comigrating (*blue*) or independently migrating fluorescent spots of mCherry-KPNA1^E448X-NES^. In (*G*), *magenta* and *green* indicate KPNA1 and DIC1, while in (*H*), *magenta* and *green* indicate KPNA1 and p150^glued^, respectively. Mean percentages from three independent experiments are shown. Measurements are derived from 16 cells, 262 fluorescent spots (*left*), and 16 cells, 563 fluorescent spots (*right*). *I*, quantification of EGFP-KPNA1^E448X^ and mKO2-Rab7a fluorescent spots. Data were collected from 10 and 17 cells, respectively, across two independent experiments. The *p*-values, calculated using Fisher's exact test for statistical analysis, are shown above. *J*, comparison of fluorescence intensities between EGFP-KPNA1^E448X^ and EGFP-KPNA1^E448X-NES^ spots comigrating with Rab7a. Measurements for EGFP-KPNA1^E448X^ were derived from 88 fluorescent spots across seven cells, while those for EGFP-KPNA1^E448X-NES^ were derived from 161 fluorescent spots across 14 cells. Data were collected from two independent experimental replicates. The *p*-value, calculated using Wilcoxon's rank sum test, is shown at the *top*.

To analyze the mobility of KPNA1^E448X-NES^ in the axonal region, mCherry-KPNA1^E448X-NES^ live imaging was performed. In contrast to KPNA1^E448X^, where the amount of axonal KPNA1 was greatly reduced, motile axonal spots were observed in DRG neurons expressing mCherry-KPNA1^E448X-NES^ (Fig. 6, *A* and *B*). Furthermore, Ciliobrevin D halted KPNA1^E448X-NES^ motility, similar to the observations for KPNA1^FL^ (Fig. 6*C*). This result indicates that KPNA1^E448X-NES^ motility depends on the molecular motor activity as observed for the KPNA1^FL^ protein (Fig. 3*D*). The mobility patterns from kymographs showed that the trajectories and velocity histograms of KPNA1^E448X-NES^ were similar to those of KPNA1^FL^, displaying relatively stable spot mobility along the axons (Fig. 6, *D* and *E*). Classification of the motility pattern of KPNA1^E448X-NES^ showed that > 50% of the spots were classified as stable or bidirectional, similar to the motility pattern of KPNA1^FL^ (Fig. 6, *D*–*F*, compared with Fig. 3*G*). These results suggest that NES addition at the C-terminus of the protein rescued axonal localization and motility, bidirectional and relatively stable motility, of the mutated KPNA1. We performed dual live imaging of EGFP-KPNA1^E448X^ and EGFP-KPNA1^E448X-NES^ with EGFP-DIC1 or EGFP-p150^glued^ (Video S10). Similar to those of mCherry-KPNA1^FL^, mCherry-KPNA1^E448X-NES^ fluorescent spots that comigrated with EGFP-DIC1/EGFP-p150^glued^ displayed more stable and bidirectional motility (Fig. 6, *G* and *H*). These results suggest that KPNA1^E448X-NES^ can directly or indirectly comigrate with the dynein/dynactin complex in axons. Furthermore, to analyze the effects of mutation in the comigration of KPNA1 with axon organelles, we observed EGFP-KPNA1^E448X^ and EGFP-KPNA1^E448X-NES^ with mKO2-Rab7a (Video S11). EGFP-KPNA1^E448X^ and mKO2-Rab7a kymographs showed that several KPNA1^E448X^ fluorescent spots comigrated with Rab7a; however, their fluorescence intensity was low (Fig. S6*A*). For KPNA1^E448X-NES^, several fluorescent spots comigrated with Rab7a, and the intensity of KPNA1^E448X-NES^ was high (Fig. S6*B*). Comigration quantification showed that the ratio of KPNA1^E448X-NES^ comigrating with Rab7a was significantly larger than that of KPNA1^E448X^ (Fig. 6*I*). Moreover, the fluorescence intensity of Rab7a-positive fluorescent spots was significantly higher in KPNA1^E448X-NES^ than in KPNA1^E448X^ (Fig. 6*J*). These results strongly suggest that adding NES to mutated KPNA1 ameliorated the abnormal localization of KPNA1, and localization of axonal KPNA1 in or near axonal endosomes was rescued.

## Discussion

**Figure 7:**
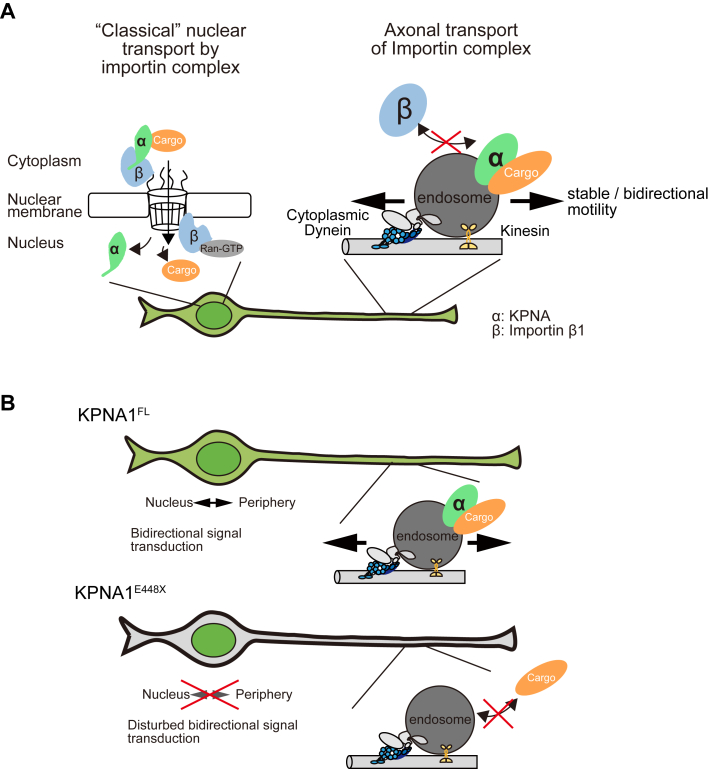
**Schematic representation of axonal KPNA1 transport**. *A*, depiction of the “classical” transport mediates by trimeric complex and the axonal KPNA transport. Axonal KPNA1 migrated within the axon independently of IPOB1. Axonal KPNA1 on endosomes shows relatively stable behavior, reflecting the mobility pattern of endosomes. Note that our model does not exclude the possibility of axonal KPNA1 in other forms. *B*, images of neurons expressing KPNA1^FL^ (*top*) or KPNA1^E448X^ (*bottom*). KPNA1^E448X^ accumulates in the nucleus, leading to a loss of axonal KPNA1 and disruption of axonal signal transduction *via* NLS-cargo transport, which may be associated with the onset of psychiatric disease.

Our results revealed a nonclassical type of protein–protein interaction among axonal importins, provided insights into the motility of axonal KPNAs, and demonstrated the effects of mutations in *Kpna1*. Our findings highlight that KPNA1 undergoes late endosome-dependent axonal transport, while the independent transport of importin α and importin β reveals distinct trafficking mechanisms between the cell body and axon. KPNA1 migrated along axons of steady-state noninjured neurons independent of IPOB1, suggesting the presence of a nonclassical transportation system for KPNA1 in axons distinct from the trimeric complex (IPOB1–cargo–KPNA). To the best of our knowledge, this is the first analysis to provide a detailed characterization of the motility of KPNAs and IPOBs in axons. Many axonal KPNAs exhibited relatively stable mobility with fluctuating movements in retrograde and anterograde directions. Upon Ciliobrevin D treatment, axonal KPNA1 movement completely ceased, in stark contrast to its dynamic mobility without the treatment. These motility patterns reflect the feature of axonal endosome/lysosome motility (31, 32), suggesting that many of axonal KPNA1 relies on axonal endosomes/lysosome for its localization and motility. Here, we propose a novel mechanism in which axonal KPNAs localize to and are transported within axons in an endosome/lysosome-dependent manner, though the possibility of the presence of different transport mechanisms for axonal KPNA1 cannot be ruled out (Fig. 7). Additionally, using peripheral neurons as a model for the axonal motility studies, a schizophrenia-related mutation in KPNA1 caused abnormal accumulation of the protein in the nucleus, along with its depletion from the cytoplasm and axons.

Our results showed that KPNAs and IPOB1 localized in the axon. The localization patterns of proteins expressed from the endogenous *Kpna1* and *Ipob1/Kpnb1* genes closely resembled those observed in gene-transfected DRG neurons (Fig. 1 and 2). KPNA1 was strongly localized in the nucleus and was present in the cytoplasm and axons of neurons. In contrast, IPOB1 was not detected in the nucleoplasm but was localized at the nuclear membrane, as well as in the cytoplasm and axons. These findings indicate that fusion with fluorescent proteins does not significantly impair protein functionality, consistent with previous findings demonstrating the presence of importins in neuronal axons (18). Previous knockout studies have demonstrated the role of KPNA1 in neuronal function (8, 9, 10). KPNA1 proteins located in both the cell body and axons likely serve essential functions in neurons. Immunoblot and immunofluorescence analyses revealed low expression levels of KPNA2, 4, and 6, with even low expression of KPNA3 in peripheral neurons (Fig. S1). In addition, our LC-MS/MS analysis identified the presence of KPNA4 in DRG neurons (Fig. 1*B*). KPNA4 plays important roles in both peripheral neurons and brain function (12, 33). Further comprehensive analyses are warranted to elucidate the distinct roles of KPNA1 and other importins in neuronal function.

Our biochemistry, immunofluorescence, and PLA results do not support axonal interaction between KPNA1 and IPOB1, suggesting that many of axonal KPNA1 and IPOB1 proteins migrate independently in noninjured static neurons. Specifically, PLA indicated an interaction between KPNA1 and IPOB1 in the cytoplasm; however, no PLA signal was observed in the axonal region (Fig. 2, *F*–*I*). These results raise the possibility that interactions between KPNA1 and IPOB1 differ between the cytoplasm and axon and that a direct interaction in the axonal region is absent. The lack of interaction may be attributed to the low amounts of IPOB1 in the axonal region, as IPOB1 protein levels in the axons of noninjured, stable neurons are very low (18). However, our immunofluorescence analysis supports the presence of a certain amount of IPOB1 in the axon (Figs. 1*H* and 2*C*). This analysis indicates that IPOB1 is present in the noninjured axon of primary cultured neurons, albeit at low abundance compared with KPNA1. Nevertheless, almost no PLA signals were observed for the combination of KPNA1 and IPOB1 in the axon (Fig. 2, *F*–*I*). Our live imaging experiments showed that a large amount of IPOB1 protein was expressed *via* the gene transfection of the *Ipob1/kpnb1* gene. However, the percentages of KPNA1-IPOB1 colocalization/comigration were low in the live imaging analysis, and most of KPNA1/IPOB1 migrated independently (Figs. 2*C*, and 4, *A* and *B*). These results support that KPNA1 and IPOB1 interact differently in the cytoplasm and axon of neurons. In our analysis, IPOB1-IP efficiently precipitated IPOB1 from the femoral nerve lysate but did not coprecipitate KPNA1 (Fig. S2*B*). Previous studies have demonstrated that KPNA1 and IPOB1 coprecipitated in pull-down experiments involving culture cells (34), indicating that interactions between KPNA1 and IPOB1 differ between the axon and the cytoplasm of the cell body. The interactions between KPNA1 and IPOB1 depend on Ran GTPase (2). LC-MS/MS results showed high expression levels of Ran and CAS exportin proteins in axons (Fig. 1*B*). Axonal Ran activity is differentially regulated under steady-state and injured conditions (35). Elevated Ran-GTP in axons may account for the low affinity between KPNA1 and IPOB1 in steady-state primary culture neurons, implying that a specific interaction exists. RanBP10 functions as a Ran-GEF (36), and axons are specialized cellular compartments with high Ran-GTP activity (37, 38, 39). The low affinity between axonal KPNA1 and IPOB1 observed in our study is consistent with these findings. Live imaging studies demonstrated that other KPNA subtypes (KPNA3, 4, and 6) exhibited mobility patterns similar to that of KPNA1, suggesting that other KPNAs are also capable of axonal transport (Fig. S3). However, the functional differences among these KPNA subtypes remain unclear and require further investigation. Although IPOB1 did not comigrate with KPNA1, it exhibited motility along axons (Fig. 3). It has been proposed that the binding of IPOB1 to the dynein complex requires a KPNA adapter (40). However, similar to KPNA1, IPOB1 displayed heterogeneity in its motility pattern, suggesting that KPNA1-independent IPOB1 transport likely reflects a distinct mechanism separate from axonal IPOB1 transport.

There may be a distinct mechanism that enables KPNAs to localize and be transported in axons. In addition to the classic pathway, KPNAs and IPOB1 can transport proteins independently of conventional heterotrimeric complexes (41). Our observation that KPNA1 and IPOB1 can function independently may potentially reflect the existence of nonclassical pathway-dependent mechanisms in axons. For example, KPNA transports calcium/calmodulin-dependent protein kinase type IV (CaMKIV) to the nucleus without the aid of IPOB1 (42). CaMKIV's functions in neurons are well-documented and crucial for long-term memory formation (43), although its direct role in the neuronal periphery is less prominent. Studies with *Caenorhabditis elegans* have suggested that transporting CaMKIV to the nucleus is crucial for behavioral adaptation (44), highlighting the importance of the CaMKIV-dependent transport system in neurons. Given the strong expression of CaMKIV in the brain and its importance in activity-dependent gene expression, it is possible that CaMKIV-regulated pathways may influence long-range axonal signaling. The IPOB1-independent transport of KPNA1 in axons might be associated with CaMKIV. However, this potential connection and the axonal transport mechanism of CaMKIV warrant further investigation.

Our biochemical assays and PLA studies did not provide evidence for a direct interaction between KPNA1 and dynein. Previous research has shown that KPNA proteins interact constitutively with the dynein intermediate chain under both steady-state and injured conditions (18). Despite this finding, the specific molecular interactions between KPNA proteins and various dynein subunits have not been thoroughly analyzed. Our KPNA1-IP or IPOB1-IP experiments did not yield strong dynein subunit precipitation. This discrepancy may be attributed to the neuron conditions used, as our analysis was conducted with stable, noninjured neurons or the efficiency of the KPNA1 antibody employed. However, our result of dynein inhibition with Ciliobrevin D suggests that the mobility of axonal KPNAs depends on the activity of molecular motors. Ciliobrevin D is known as a dynein inhibitor; it is reported that anterograde and retrograde motility are interdependent, and inhibition of dynein results in reduced motility in both directions (26, 45). The observed inhibition of KPNA1 mobility by Ciliobrevin D indicates that the axonal motility of KPNAs relies on the activity of dynein and kinesin. Therefore, KPNAs may interact with dynein, not as a dynein adaptor but as an indirect binding partner or cargo of dynein and kinesin.

Herein, we propose a new mechanism underlying KPNA1 localization and transport dependent on axonal endosomes/lysosomes (Fig. 7). Immunofluorescence and PLA studies suggested that KPNA1 did not proximally localize with cytoplasmic dynein or kinesin motors. However, these studies indicated the vicinal localization of KPNA1 and Rab7a (Fig. 2, *F*–*I*). The inability to confirm the coprecipitation of KPNA1 and molecular motors through conventional immunoprecipitation suggests that the interaction between KPNA1 and motors is dependent on endosomes. Our analysis showed that the motility of KPNA1 is heterogeneous, with most particles exhibiting stable with fluctuating motility and frequently switching directions. Endosomes interact with both kinesins and dynein motors (46). Thus, the dynamics of KPNA1 could be explained by the motility of endosomes in axons *via* the tug-of-war or the cooperative motility of dynein and kinesin on endosomes (32, 47, 48). However, we must also consider the possibility that there exists a transport mechanism for KPNA1 that does not depend on axonal endosomes/lysosomes and that the complex formation of KPNA1 in axons may not be uniform.

Our analysis showed that KPNAs are transported bidirectionally, not limited to retrograde directions. Many of KPNA1 showed that most of axonal KPNA1 were stable on axon (Fig. 3, *D*–*G*). Previous research has reported that over 50% of endosomes exhibit stable mobility in axons (31). Our analysis of KPNA1 demonstrated similar motility patterns. The motility of axonal importins was relatively stable yet fluctuating but not completely static. The differences in kymographs between Ciliobrevin D–treated and untreated samples strongly suggest that the fluctuating movement of KPNA1 depends on molecular motors. Such motility patterns can be explained by the dynamics of endosomes. While retrograde motility was anticipated based on previous findings that indicated importin–dynein interactions in neuronal peripheries, such as axons or synapses (18, 21, 49), our results suggest that motility is not confined to retrograde directions. This bidirectionality can be attributed to the dependence on the endosome/lysosome motility. Our study was limited to the steady-state axons of DRG neurons. Further investigation is necessary to determine whether external signals can affect the motility of endosomes and KPNAs. Moreover, axonal transport is a complex system involving various cargos, and it remains possible that KPNA1 is associated with other transport systems.

The observed association between KPNA1 and late endosomes/lysosomes raises compelling questions regarding its functional importance. While this study did not yield conclusive answers, several potential roles for this interaction can be hypothesized: (1) Axonal transport: KPNA1 might employ endosomal "hitchhiking" for efficient axonal movement, analogous to mechanisms documented for mRNAs or RNA granules (50, 51). This could enable swift redistribution of KPNA1 in response to cellular demands. (2) Protein turnover regulation: The connection with late endosomes and lysosomes may represent a mechanism for modulating KPNA1 levels, possibly involving regulated degradation to precisely control nuclear import processes. (3) Signaling pathway involvement: KPNA1 might participate in endosome-mediated signaling cascades, bridging cytoplasmic events with nuclear responses. (4) Recycling mechanism: This interaction could be part of a recycling pathway for KPNA1, facilitating its reuse in multiple cycles of nuclear import. Interestingly, axonal NR1, a subunit of NMDAR, is actively transported in axon (52). Considering the interaction between KPNA and NMDAR at synapses (21), interaction between KPNA1 and NMDAR proteins in axons is intriguing. Further research is necessary to elucidate the exact nature and significance of these potential functions.

Our analysis revealed that the schizophrenia-related E448X mutation caused abnormal accumulation of KPNA1 in the nucleus, and the addition of a short sequence restored axonal localization and motility. In this analysis, we took advantage of DRG neurons, which have no dendrite structure but bifurcating axons to easily distinguish anterograde and retrograde directions of axons. Our model was restricted to peripheral neurons, and whether the effects of mutations necessarily reflect their function in the CNS remains unclear. Further studies on the CNS are required to fully understand the implications of our findings for the onset of diseases such as schizophrenia. Notably, *Kpna1* is highly expressed in both CNS and peripheral neurons, and the mutation observed in CNS disrupted KPNA1 localization in peripheral neurons. While the precise relationship between the mutation and schizophrenia remains unclear, the mislocalization of KPNA1 and/or loss of its axonal transport may be linked to the onset of the disease.

Mutations in the *Kpna1* gene and its mislocalization in the periphery of neurons may be related to the onset of diseases. Several genes associated with synaptic functions have been linked to schizophrenia (53). Notably, the deregulation of the synapto-nuclear shuttling of postsynaptic density proteins is associated with schizophrenia (54). The disruption of NMDAR transport by microtubule motor proteins contributes to the development of schizophrenia-like phenotypes in mice (14). KPNA functions as an adapter of cargo that transmits signals from synapses to the nucleus (43) and directly binds to NMDAR (21). Mislocalized KPNA1 likely downregulates signal transduction, inducing the onset of diseases.

Augmenting the export of KPNA1 protein from the nucleus by introducing a short sequence into the mutated *Kpna1* gene in the nucleus restored its mobility within the axon, which has a significant clinical implication. The addition of NES to the mutated protein rescued the localization and mobility of axonal KPNA1, likely *via* the export activity mediated by CRM1. Moreover, KPNA1^Δ^^N^ did not significantly affect nuclear accumulation, as previously reported in cultured cells (55). However, the combination of the ΔN mutation and E448X (KPNA1^Δ^^N-E448X^) prevented abnormal protein accumulation in the nucleus. These results indicate that abnormal KPNA1 accumulation in the nucleus requires the activity of IPOB1, which binds to the IBB domain in the N-terminus of KPNA1. While our results indicate that axonal KPNA1 migrates independently of IPOB1, its nuclear localization depends on IPOB1 activity. The only difference between KPNA1^E448X^ and KPNA1^E448X-NES^ was the short 12-residue; therefore, it is unlikely that KPNA1^E448X^ lost its axonal localization ability. Instead, anomalous KPNA1 accumulation owing to C-terminal deletion hindered the distribution of KPNA1 to the axon and caused the loss of axonal KPNA1.

In conclusion, our findings provide novel insights into the dynamics of axonal importins and changes in localization associated with schizophrenia-related mutations. The mobility patterns of axonal KPNAs and IPOB1 deviated from the expected motility of classical importins and the binding partners of KPNAs and IPOB1 diverged from the anticipated factors, suggesting their independent transport within the axon. These results expand our understanding of the multifaceted roles played by importins. While our study primarily focused on the peripheral nervous system, further analysis is needed to elucidate the connection between these findings and CNS development, as well as the onset of psychiatric disorders. Ameliorating localization abnormalities by adding short sequences is a promising strategy for developing effective therapeutic approaches. Further investigations into the shared molecular mechanisms underlying the onset of psychiatric and neurodegenerative disorders, including schizophrenia, remain warranted.

## Experimental procedures

### Animals

All mouse and rat experimental procedures were approved by the Regulations for Animal Research [R04022] and the Safety Management Committee for Genetic Recombination Experiments [R03020] at the University of Fukui. All methods were performed in accordance with the ARRIVE guidelines (https://arriveguidelines.org).

### Primary culture of DRG neurons

DRGs were collected from postnatal mice (C57BL/6J; P2–3), dissociated as previously described (25, 56). DRGs were collected in Hank’s Balanced Salt Solution (HBSS; H8264, Sigma-Aldrich) until further use. Ganglia were treated with 0.5 mg/ml of collagenase (C0130, Sigma-Aldrich) in 3 ml of HBSS for 15 min at 37 °C. After spinning down the ganglia, the pellet was suspended in 3 ml of HBSS containing 0.25% trypsin solution (15090-046, Gibco) and 0.2% DNase I (DN25, Sigma-Aldrich) for 20 min at 37 °C. Trypsin digestion was terminated by adding 0.5 ml of fetal bovine serum (FBS) and the cells were dissociated *via* pipetting. Debris was removed using a cell strainer (ϕ70 μm, 352350, Falcon, Corning).

The cells were washed twice with 6 ml of HBSS and suspended in Dulbecco’s modified Eagle’s medium (DMEM, High Glucose) with high glucose with L-glutamine and phenol red (DMEM; 044-29765, Fujifilm) containing 10% FBS (HyClone, Cytiva) and 200 ng/ml of 2.5S murine nerve growth factor (mNGF) (G5141, Promega), plated onto poly-D-lysine–coated dishes (P35GC-0-14-C/H, MatTek), and cultured for 24 h. The culture medium was then replaced with either fresh DMEM containing 10% FBS and 200 ng/ml mNGF or Neurobasal medium (21103049, Gibco) supplemented with L-glutamine (A2916801, Gibco) and B27 supplement (17504044, Gibco) to remove dead cells and debris. The cultures were maintained at 37 °C with 5% CO_2_ several days until use.

### Cell culture

NIH3T3 cells were obtained from the Japanese Collection of Research Bioresources (JCRB0615, lot number 11202013; National Institutes of Biomedical Innovation, Health and Nutrition). The cells were cultured in DMEM supplemented with 10% FBS. The cultures were maintained at 37 °C with 5% CO_2_ and passaged every 3 days.

### Preparation of lysate

To prepare mouse nerve lysate for immunoblotting or LC-MS/MS, DRG were collected from postnatal mice (C57BL/6J; P2–3 for immunoblotting, P4–5 for LC-MS/MS). For rat femoral nerve lysate, adult female Sprague-Dawley rats (3 months old) were anesthetized *via* inhalation of 4% isoflurane. Both sides of the femoral nerve root were surgically exposed and excised.

The DRGs or excised rat femoral nerve roots were washed with PBS (PBS; 137 mM NaCl, 2.7 mM KCl, 10 mM Na_2_HPO_4_, and 1.8 mM KH_2_PO_4_) and subjected to protein extraction with homogenization in a lysis buffer (20 mM Hepes-KOH, pH 7.3, 110 mM CH_3_COOK, 5 mM CH_3_COONa, 2 mM Mg(CH_3_COO)_2_, 1 mM EGTA, 1 mM DTT, and 1 μg/ml each of aprotinin, leupeptin, and pepstatin). Additionally, 0.1% Triton X-100 was added to the buffer, except for the buffer used in the sucrose gradient centrifugation experiment. For LC-MS/MS, extracted DRGs were dissociated by treatments with collagenase and trypsin, as described in the primary culture methods. Following homogenization, the lysates were centrifuged at 20,000×*g* at 4 °C for 15 min in the LC-MS/MS experiment and for 30 min in other experiments. The resultant supernatants were collected and used as the lysate.

To prepare mouse whole brain lysate for immunoprecipitation and immunoblotting, the whole brain was obtained from a P1 C57BL/6J mouse pup. The cerebellum and mid brain were removed and only the cerebrum was collected, which was washed with PBS and homogenized with 0.5 ml of lysis buffer. The lysates were centrifuged at 20,000×*g* at 4 °C for 30 min; the resultant supernatants were collected and used as the lysate.

### Sucrose density gradient centrifugation

For the sucrose density gradient centrifugation, rat femoral lysate, which was obtained without 0.1% Triton X-100 addition, was fractionated in 12 ml of 5 to 20% sucrose gradient in the lysis buffer. Centrifugation was performed at 18,000×*g* for 18 h at 4 °C in an RPS40T rotor with a Hitachi CP80WX ultracentrifuge (Hitachi, Ltd). Fractions of 1 ml were collected from the tube bottom and prepared for SDS-PAGE by adding 200 μl of the 5 × SDS-sample buffer (62.5 mM Tris–HCl, pH 6.8, 10% glycerol, 2% SDS, 5% 2-mercaptoethanol, and 0.02% Bromophenol blue), following which the mixture was boiled at 98 °C for 3 min. The fractions were analyzed *via* immunoblotting.

### Immunoprecipitation

Rat femoral nerve and mouse brain lysates were obtained, as described in the section above. Lysate protein concentration was adjusted to 1 mg/ml. For the rat femoral nerve lysate, 5 μg of anti-KPNA1 (anti-importin α5, 18137-1-AP, Proteintech) or anti-IPOB1 (anti-IPOB1 antibody, 10077-1-AP, Proteintech) was mixed with the lysate and incubated at 4 °C for 1h. For the mouse brain lysate, 5 μg of anti-KPNA1 (H00003836-A02, Abnova), anti-KPNA1 (114-E12, Thermo Fisher Scientific), or anti-DIC antibody (MAB1618, Sigma-Aldrich) were mixed and incubated with the lysate. The same amount of nonimmune rabbit serum was used as the control. Protein A Mag Sepharose Xtra beads (Cytiva) and 50 μl of 10% slurry were washed with the lysis buffer three times and added to the mixture of the antibodies and lysate. The mixture was rotated at 4 °C for 1 h. The beads were washed four times with the lysis buffer and resuspended in 50 μl of the lysis buffer. To elute the bound proteins, 12.5 μl of 5 × SDS-PAGE sample buffer was added to the beads, following which it was heated at 95 °C for 3 min. The resultant supernatants were analyzed *via* immunoblotting.

### Proteomic analysis

For the LC-MS/MS analysis, we used 6.8 μg of mouse DRG lysate. DRG lysates were mixed with SDS-sample buffer, boiled at 95 °C for 3 min, and subjected to SDS-PAGE (5–20% e-PAGEL, ATTO). The top region of gel containing concentrated samples was excised at a length and thickness of 2 to 3 and 1 mm, respectively. The gel slices were subjected to in-gel digestion as previously described (57). In particular, the gel slices were destained in 50 μl of 25 mM NH_4_HCO_3_/50% acetonitrile for 30 min at room temperature, and the supernatants were discarded. This procedure was repeated three times until the gel slides were completely destained. The gel slices were incubated in 200 μl of 100% acetonitrile for 10 min and dried. Dried gel slices were reduced with 10 mM DTT/50 mM NH_4_HCO_3_ at 56 °C for 1 h. After discarding the DTT solution, 200 μl of 55 mM iodoacetamide/50 mM NH_4_HCO_3_ was added and the mixture was incubated for 45 min at room temperature in the dark. The solutions were discarded and the gel slices were shaken in 100 μl of 25 mM NH_4_HCO_3_/50% acetonitrile for 30 min at room temperature. This procedure was repeated three times. Gel slices were shaken in 200 μl of acetonitrile for 10 min at room temperature and dried. The dried gel pieces were treated with 25 μl of 2.5 μg/ml trypsin (Sequencing Grade Modified Trypsin, V5113, Promega) at 37 °C overnight. Following digestion, peptides were desalted in 125 μl of 25 mM NH_4_HCO_3_/50% acetonitrile and shaken for 120 min at room temperature; 2.5 μl of formic acid was added and the mixture was shaken for 20 min and dried. Prior to analysis, 50 μl of 0.1% formic acid was added, dissolved, and kept at 4 °C until further use.

Protein digests were loaded onto an EASY-nLC 1000 LC system equipped with an EASY-Column C18-A1 5-μm, 100-μm inner diameter × 20-mm column equilibrated with 0.1% formic acid and eluted with a linear gradient of acetonitrile (0–50%) in 0.1% formic acid at a flow rate of 200 nl/min. The eluted peptides were then separated on an analytical column (C18 capillary tip column, 75-μm inner diameter × 120 mm; Nikkyo Technos) with a spray voltage of 1.5 kV. Peptide ions were detected using a Thermo Fisher Scientific LTQ Orbitrap Elite mass spectrometer in data-dependent acquisition mode with the Xcalibur software (version 2.2; Thermo Fisher Scientific). Full-scan mass spectra were acquired in MS over 400 to 2000 m/z with a resolution of 60,000. The 10 most intense precursor ions were selected for collision-induced fragmentation in the linear ion trap at a normalized collision energy of 35%. Dynamic exclusion was employed within 90 s to prevent repetitive selection of peptides.

Raw MS files were analyzed using MaxQuant version 2.0.1.0 (58). The MS/MS spectra were searched using the Andromeda search engine (59) against the database constructed using the amino acid sequences of *Mus musculus* from UniProt (https://www.uniprot.org/proteomes/UP000000589). For searches, carbamidomethylation of cysteine was set as the fixed modification, while methionine oxidation and N-terminal acetylation were set as variable modifications. The false discovery rate was set to 0.01 for peptide and protein identifications. The label-free quantification algorithm was used to rank the absolute abundance of different proteins within a single sample (60).

### SDS-PAGE and immunoblot analysis

For the immunoblot analysis, the following antibodies were used: anti-KPNA1 (1:1000, 18137-1-AP, Proteintech), anti-IPOB1 (1:1000, 10077-1-AP, Proteintech), anti-KPNA3 (1:1000, 67892-1-Ig, Proteintech), anti-KPNA4 (1:1000, 12463-1-AP, Proteintech), anti-KPNA6 (1:1000, SCE390055 (E-11), Santa Cruz Biotechnology, Inc.), and anti-DIC (1:1000, MAB1618, Sigma-Aldrich) and anti-Kinesin heavy chain (1:1000, MAB1614, Sigma-Aldrich). Both DIC and KHC antibodies are general anti-IC or anti-HC antibodies, respectively, rather than identifying specific protein names (this applies to immunofluorescence as well). To prepare the samples, proteins were treated with SDS sample buffer and boiled at 98 °C for 3 min and separated using 12% SDS-PAGE gel and transferred to polyvinylidene difluoride membranes. Subsequently, the membranes were blocked with 7.5% skim milk in Tris-based saline containing 0.01% Tween-20 and incubated with primary antibodies in CanGetSignal solution 1 (NKB-101, Toyobo) for 1 h at room temperature. After washing with Tris-based saline containing 0.01% Tween-20 five times, the membranes were incubated with secondary antibodies (HRP-conjugated anti-rabbit or anti-mouse antibodies; 115-035-072 or 111-035-144 respectively, Jackson ImmunoResearch Labs) at a dilution of 1:1000 in CanGetSignal solution 2 for 1 h at room temperature. Following washing five times, the blots were developed with immunostar Z (FUJIFILM Wako Pure Chemical Corporation) as per the manufacturer’s instructions, and signals were detected using the LAS-4000 mini imager (FUJIFILM).

### Preparation and transfection of cDNA constructs

The vectors cpEGFP-mKPNA1, pmCherry-mKPNA1, pEGFP-hIPOB1, and cDNA for KPNA3, 4, and 6 were kindly provided by Dr Yoichi Miyamoto. The cDNAs of mKPNA3, 4, and 5 were subcloned into pmCherry-C1. For pmNeonGreen-hIPOB1, hIPOB1 was subcloned from pEGFP-hIPOB1 into pmNeonGreen-C1. A deletion mutant of KPNA1 was constructed with mCherry-KPNA1-C1 using the In-Fusion HD Cloning kit (Takara Bio). Plasmids pEGFP-DIC1 and pEGFP-p150^glued^ were prepared in our previous study (24). Plasmid mKO2-Rab7a-7 was kindly provided by Dr Michael Davidson (Addgene plasmid 57,893; http://n2t.net/addgene:57893, RRID: Addgene_57893).

For transfection, 1 μg of plasmid DNA was transfected simultaneously with the preparation of primary cultured DRG neurons using the Neon transfection system (Thermo Fisher Scientific) or Lipofectamine 3000 (Thermo Fisher Scientific) as per the manufacturer’s instructions. After 24 h of incubation with the transfection reagent at 37 °C/5% CO_2_, the culture medium was replaced with fresh D-MEM/10% FBS/mNGF solution or Neurobasal/L-glutamine/B27 supplement medium and kept at 37 °C/5% CO_2_ until use.

### Live-cell imaging and FRAP

Live-cell imaging and FRAP analysis was conducted with DIV2–4 of DRG neurons after 24 to 48 h of transfection. DRG neurons were observed using an IX83 microscope equipped with an FV1200 confocal microscopy system with 60 × objective (PlanApo N, N.A 1.42; Olympus) and a stage-top incubator at 37 °C and 5% CO_2_ (INUG2F-IX3W, Tokai Hit). For single channel or dual-color live imaging, EGFP, Venus, and mNeonGreen signals were obtained with a laser at wavelength of 473 nm and mCherry with a laser at a wavelength of 559 nm. Each image was taken at a rate of three frames/s. Image processing, kymograph generation, and measurement of recorded images was conducted using *Fiji/ImageJ* (version 1.54c) and R/Rstudio (version 4.2.3) (61). Trajectories of fluorescent spots were obtained from the semi-manual tracing of kymograph and transformed to *x*, *t* coordinates using *Bohboh* software (ver. 4.90 J, Bohbohsoft). The average velocities of fluorescents spots were analyzed by manually tracing the kymographs with the segmented line tool of *Fiji/ImageJ*, and *x*, *t* coordinates were obtained. The slope of each coordinate of trajectories obtained from the kymographs of 60 s time series and the average, minimal, and maximal velocities were analyzed. The average values were obtained by weighting the durations of each velocity segment.

For comigration analysis, kymographs were generated using *Fiji*/*ImageJ* to visualize the motility of these fusion proteins along the axon. The comigration of each fluorescent spot was compared by overlaying the green and red fluorescent channels and judged by matching each trace in kymographs. The percentage of colocalization was determined by counting all manual traces from the green and the red channels.

For FRAP analysis, a rectangular area with a length of 80 to 100 μm was bleached using 100% transmittance of a 473 or 567 nm wavelength laser with the FV1200 confocal microscopy system (Olympus). The time series of fluorescence recovery was obtained from regions of interest on the proximal and distal ends of bleached regions (^∼^10 μm in length) at 0.25 s intervals 2.5 s prior to photobleaching and for 60 s after photobleaching. FRAP curves were fitted to the single exponential equation, f(t)=A(1−exp(−τt)), where t, A, and τ represent the time, mobile fraction, and time constant, respectively. The half-life (t_1/2_) was defined as $\frac{\ln\left(2\right)}{\tau }$.

### Immunofluorescence

For immunofluorescence detection, the following antibodies were used: anti-KPNA1 (1:200, 18137-1-AP, Proteintech), anti-IPOB1 (1:200, 10077-1-AP, Proteintech), anti-KPNA3 (1:200, 67892-1-Ig, Proteintech), anti-KPNA4 (1:200, 12463-1-AP, Proteintech), anti-KPNA6 (1:200, SCE390055 (E-11), Santa Cruz Biotechnology), anti-DIC (1:200, MAB1618, Sigma-Aldrich), anti-KHC (1:200, MAB1614, Sigma-Aldrich), anti-Tuj1 (1:1000, GT886, GeneTex), and anti-Rab7a (1:200, ab126712, Abcam). Anti-p150^glued^ antibody is from the previous study (25). The primary culture of DRG neurons was fixed with ice-cold 100% methanol for 20 min at −30 °C (Fig. 1, *C–H*, and Fig. 2, *A* and *C* or fixed with 4% paraformaldehyde in PBS for 15 min at 37 °C (Fig. S1). The cells fixed with 4% paraformaldehyde were permeabilized with 0.1% Triton X-100 in PBS for 15 min at 25 °C. Permeabilized cells were blocked with 5% bovine serum albumin (BSA)/0.05% Triton X-100/PBS for 1 h at room temperature. Primary antibodies were suspended in 5% BSA/0.05% Triton X-100/PBS and incubated for 1 h at room temperature. Cells were washed five times with 0.05% Triton X-100/PBS and incubated with secondary antibodies (Alexa 488-conjugated or Alexa 555-conjugated, anti-mouse and anti-rabbit antibodies, Invitrogen) at a dilution of 1:1000 in 5% BSA/0.05% Triton X-100/PBS for 1 h at room temperature. After washing twice with 0.05% Triton X-100/PBS, the cells were incubated with 10 μg/ml Hoechst 33342 for 10 min at room temperature, followed by three additional washes.

The specimens were observed using an IX83 microscope equipped with an FV1200 confocal microscopy system with 60 × objective (PlanApo N, N.A 1.42; Olympus). Immunofluorescence signals between two antigens were quantified using Pearson’s correlation coefficient calculated with the JACOP plug-in in *Fiji/ImageJ*. XYZ stack images of cells were used for the analysis to ensure accurate comparisons.

### Proximity ligation assay

PLA was performed using Duolink *In Situ* Detection Regents Red according to the Duolink manufacturer’s instructions (DUO92008, Sigma-Aldrich). DRG neurons at DIV3 or NIH3T3 cells were fixed and permeabilized with 100% methanol for 20 min at −30 °C. The cells were blocked with the Duolink blocking solution for 1 h at 37 °C. Subsequently, the cells were incubated with the primary antibodies for 1 h at room temperature. The following combinations of antibodies were used in the Duolink antibody diluent: anti-KPNA1 (host: rabbit), anti-EGFP (mouse), anti-KPNA1 (rabbit), and anti-IPOB1 (mouse), anti-Rab7a (1:200, ab126712, Abcam), anti-IPOB1 (1:200, 10077-1-AP, Proteintech), anti-DYNCH1 (cytoplasmic DHC) (1:400, 12345-1-AP, Proteintech), anti-p150^glued^ (raised in laboratory) (25), anti-Kinesin heavy chain (1:200, MAP1614 Sigma-Aldrich). Additionally, neurons were probed with anti-microtubule–associated protein (MAP2) (raised in chicken, 1:10,000, ab5392, Abcam) to detect neurons from the primary culture.

The cells were washed three times with the Duolink wash buffer A and incubated with the probe (anti-rabbit Plus and anti-mouse Minus) and secondary antibody for anti-MAP2 (Alexa 488–conjugated anti-chicken IgY antibody, A32931, Invitrogen) for 1h at 37 °C. The cells were washed three times with the Duolink wash buffer A, and ligation was performed with Duolink ligase in 1x ligation buffer for 30 min at 37 °C. Subsequently, the cells were washed three times with Duolink wash buffer A and subjected to amplification with Duolink polymerase in an amplification buffer for 100 min at 37 °C. The cells were washed three times with Duolink 1x wash buffer B and with 0.01 x wash buffer B for 1 min. The slides were mounted with Duolink *in situ* mounting medium with DAPI and imaged using the FV1200 confocal microscope. Quantification was performed by measuring the value of integrated intensity per area (μm^−2^) using *Fiji/ImageJ*.

### Statistical analysis

Statistical analyses and graph creation were performed using R (version 4.3.0) and the ggplot2 package (62), respectively. The specific statistical tests used are described in the figure legends. A *p-*value of < 0.05 was considered statistically significant.

## Data availability

The authors affirm that the data substantiating the results of this study can be found within the paper and its supplementary information files. Upon reasonable request, the corresponding author can provide access to the data. Source data accompany this paper. For materials and correspondence: Masami Yamada E-mail: yamadama@u-fukui.ac.jp.

## Supporting information

This article contains supporting information.

## Conflicts of interest

The authors declare that they have no conflicts of interest with the contents of this article.

## Supporting information

Supplemenatry Materials

Supplemenatry Figures

Supplemenatry Table
