# Supplementary material for: Direct observation of importin α family member KPNA1 in axonal transport with or without a schizophrenia-related mutation
Source: J Biol Chem. 2025 Feb 24;301(4):108343. doi: 10.1016/j.jbc.2025.108343 (PMC11982482; doi:10.1016/j.jbc.2025.108343)
Supplement: Supplemenatry Materials [file mmc1.docx]

**Supplementary Movie Legends**

**Video S1**

A representative Z-stack image of an NIH3T3 cell subjected to PLA with KPNA1 and IPOB1.

**Video S2**

A representative Z-stack image of a DRG neuron subjected to PLA with KPNA1 and IPOB1.

**Video S3**

Representative live imaging video of EGFP-KPNA1 expression in a dorsal root ganglion (DRG) neuron for FRAP analysis. The white rectangle indicates the photobleaching site. Time stamps in the corner are displayed as “min:sec.”

**Video S4**

(Top) Representative live imaging video of mCherry-KPNA1 expression in a DRG neuronal axon. Time stamps in the corner are displayed as “min:sec”. (Bottom) Representative live imaging video of mCherry-IPOB1 expression in a DRG neuronal axon. Time stamps in the corner are displayed as “min:sec”.

**Video S5**

(Top) Representative live imaging video of non-fused mCherry monomer expression in a DRG neuronal axon transfected with mCherry-C1. Time stamps in the corner are displayed as “min:sec”. (Middle) Representative live imaging video of mCherry-KPNA1 expression in a DRG neuronal axon treated with 25 µM of a dynein inhibitor Ciliobrevin D. Time stamps in the corner are displayed as “min:sec”. (Bottom) Representative live imaging video EGFP-DIC1 expression in a DRG neuronal axon. Time stamps in the corner are displayed as “min:sec”.

**Video S6**

Simultaneous presentation of two representative dual-color live imaging videos showing EGFP-KPNA1 and mCherry-IPOB1 expression (Tops), and mNeonGreen (mNG)-IPOB1 and mCherry-KPNA1 expression (Bottoms) in DRG axons. Time stamps in the corner are displayed as “min:sec.”

**Video S7**

Representative live imaging video of dual-color live imaging showing EGFP-DIC1 and mCherry-KPNA1 expression in a DRG neuron. Time stamps in the corner are displayed as “min:sec”.

**Video S8**

Representative live imaging video of dual-color live imaging showing EGFP-p150^glued^ and mCherry-KPNA1 expression in a DRG neuron. Time stamps in the corner are displayed as “min:sec”.

**Video S9**

Simultaneous presentation of two representative dual-color live imaging videos showing Venus-KPNA1 and mKusabira-orange (mKO2)-Rab7a expression (Tops), and EGFP1-KPNA1 labeled with LysoTracker Red (Bottoms). Time stamps in the corner are displayed as “min:sec.”

**Video S10**

Simultaneous presentation of two representative dual-color live imaging videos showing EGFP-KPNA1^E448X^ and EGFP-p150^glued^ expression (Tops), and EGFP1-KPNA1^E448X-NES^ and EGFP-p150^glued^ expression (Bottoms). Time stamps in the corner are displayed as “min:sec.”

**Video S11**

Simultaneous presentation of two representative dual-color live imaging videos showing EGFP-KPNA1^E448X^ and mKO2-Rab7a expression (Tops), and EGFP1-KPNA1^E448X-NES^ and mKO2-Rab7a expression (Bottoms). Time stamps in the corner are displayed as “min:sec.”

**Supplementary Table 1**

A list of mouse DRG lysates showing LFQ intensity values and Z-score transformed values obtained via LC-MS/MS.
