# Supplementary material for: Direct observation of importin α family member KPNA1 in axonal transport with or without a schizophrenia-related mutation
Source: J Biol Chem. 2025 Feb 24;301(4):108343. doi: 10.1016/j.jbc.2025.108343 (PMC11982482; doi:10.1016/j.jbc.2025.108343)
Supplement: Supplemenatry Figures [file mmc2.pdf]

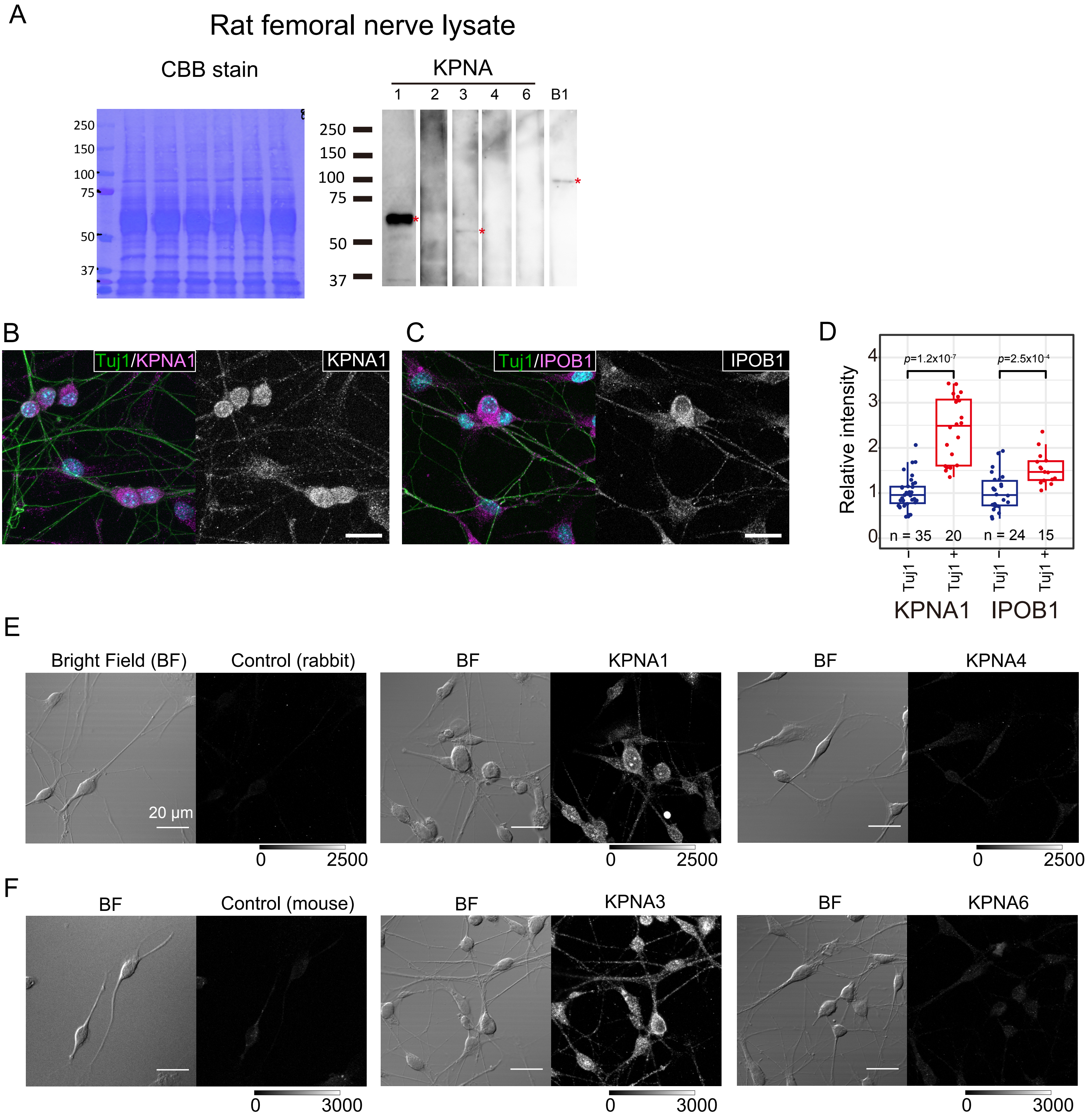

Fig. S1

(A) CBB-stained protein extract from rat femoral nerve used as a loading control. On the right: each lane on the left membrane was cut and probed with different antibodies, which were simultaneously detected. Asterisks indicate the bands detected by the antibodies. 1, 2, 3, 4, 6: KPNA1/2/3/4/6. B1: IPOB1.

(B, C) Immunofluorescence of dorsal root ganglia (DRG) neurons probed with (B) Tuj1 and KPNA1 antibodies or (C) IPOB1 antibodies. On the left: merged image of nucleus staining with Hoechst, anti-Tuj1, anti-KPNA1 (B) or IPOB1 **(C)**. On the right: single-channel images for KPNA1 (B) or IPOB1 (C). Scale bar: 20 µm.

(**D**) Comparison of intensity values of KPNA1 or IPOB1 in the cell bodies of Tuj1-negative (−) or Tuji-positive (+) cells. Intensity values were normalized using the mean intensity of Tuj1-negative cells. KPNA1 and IPOB1 intensity values were significantly higher in Tuj1 (+) cells than in Tuj1 (−) cells. The *p*-values are indicated in the graph. Welch' s *t*-test was used with Holm' s method for multiple comparison correction. The cell numbers are shown at bottom.

(**E**) Immunofluorescence of mouse DRG neurons probed with rabbit antibodies: The secondary antibody only (rabbit control; left) anti-KPNA1 (center), and anti-KPNA4 (right).

(**F**) Immunofluorescence of mouse DRG neurons probed with mouse antibodies: second antibody only (mouse control; left), anti-KPNA3 (center), and anti-KPNA6 (right).

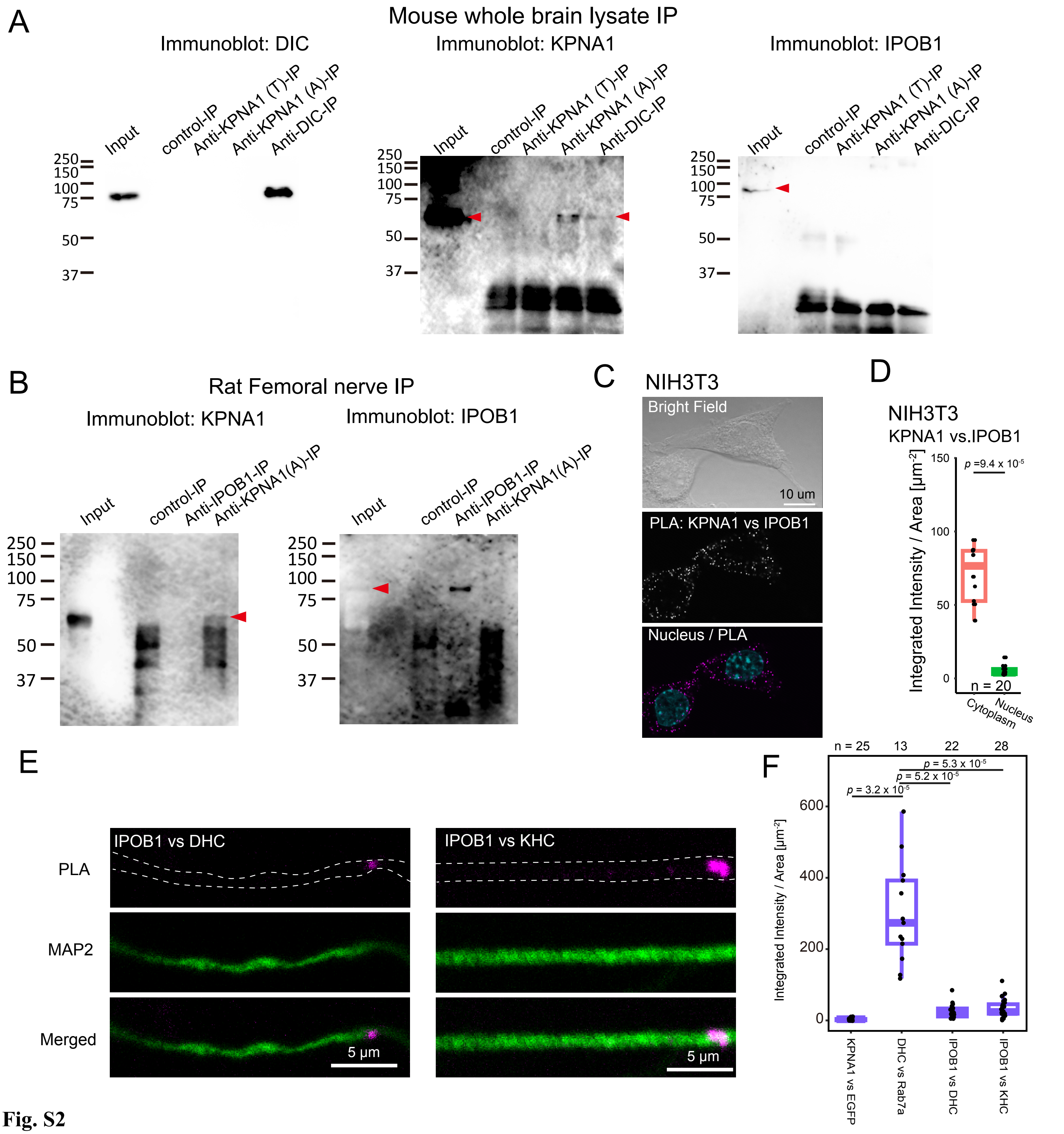

Fig. S2

(A) Immunoblotting of immunoprecipitated (IP) samples from mouse whole-brain lysates. Control: IP sample with non-immune rabbit antibodies. Anti-KPNA1 (T): IP sample with anti-KPNA1 antibody from ThermoFisher (E114-E12). Anti-KPNA1 (A): IP sample with anti-KPNA1 antibody from Abnova (H00003836-A02). Blots were probed with anti-DIC, anti-KPNA1, and anti-IPOB1 antibodies. **(B)** Immunoblotting of IP samples from rat femoral nerve lysates using control IP, anti-IPOB1 IP, and anti-KPNA1 IP antibodies (Abnova). Blots were probed with anti-KPNA1 and anti-IPOB1 antibodies. WB: Western blot. **(C)** Images of PLA dots showing interactions between KPNA1 and IPOB1 in NIH3T3 cells. Brightfield images, images of PLA dots, and merged images of PLA dots with Hoechst-stained nuclei (labeled as Nucleus) are shown. (**D**) Quantification of integrated intensity/area (A.U./μm²) of PLA dots representing interactions between KPNA1 and IPOB1 in NIH3T3 cells. PLA intensity values in the cytoplasm and nucleus were compared. The *p*-value from Welch' s *t*-test is indicated above. N = 20 (cell number). (**E**) Magnified images of DRG neuron axons examined using PLA experiments with IPOB1 and DHC, as well as IPOB1 and KHC. Axonal regions are outlined with white dashed lines. Scale bar: 5 μm. (**F**) Quantification of integrated intensity/area (A.U./μm2) of PLA dots in axonal regions. Antibody combinations are indicated at the bottom. Negative and positive controls (KPNA1 and EGFP, DHC and Rab7a) are the same as Fig. 3I. The *p*-values from Welch' s *t*-test with Holm' s correction for multiple comparisons are indicated above. Cell numbers are 13 – 28 from two times experiments.

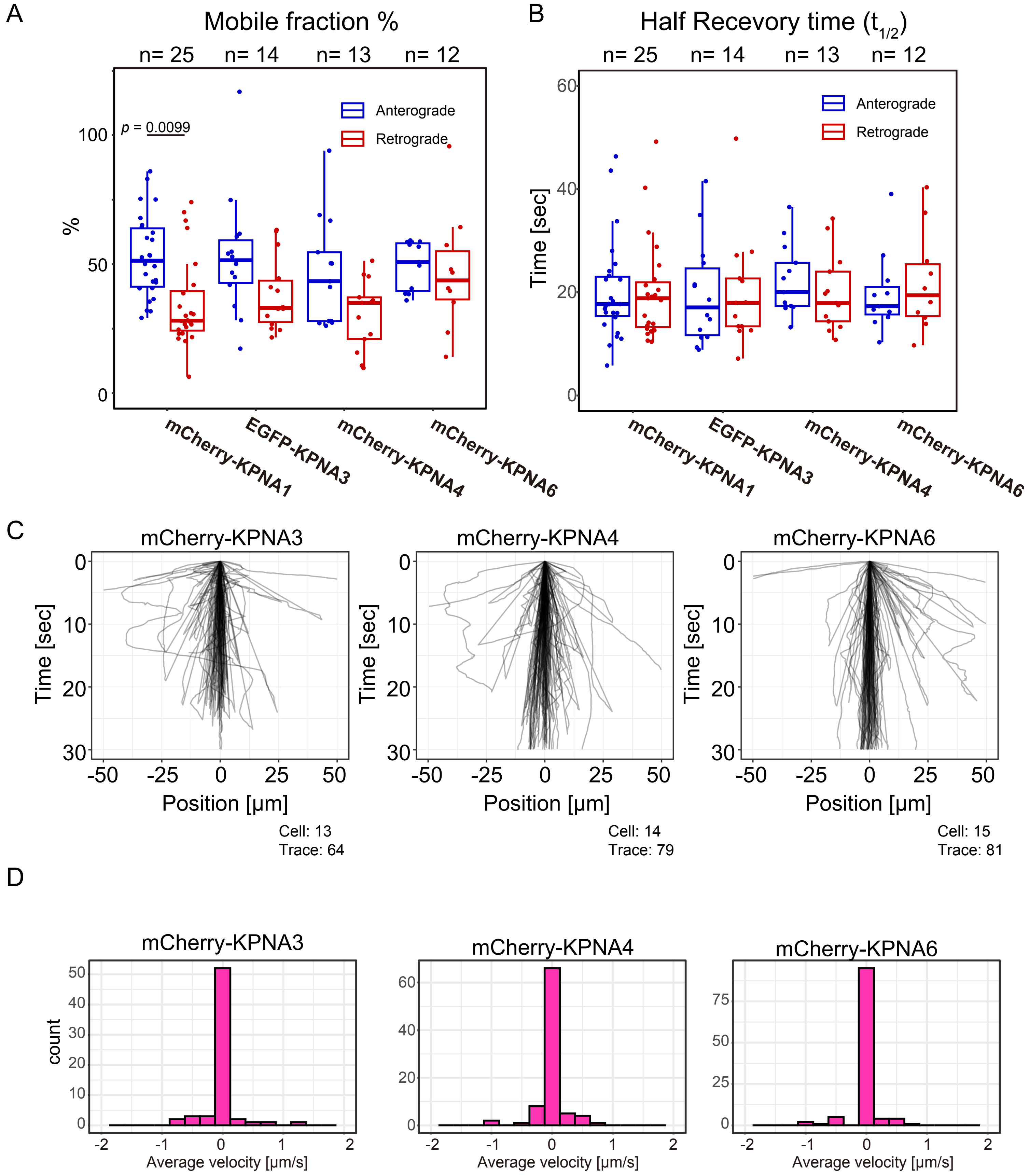

Fig. S3

(A, B) Comparison of parameters obtained from FRAP analysis for mCherry-KPNA1, EGFP-KPNA3, mCherry-KPNA4, and mCherry-KPNA6. (A) Mobile fraction. **(B)** Half-time for fluorescence recovery. The *p* value from Wilcoxon' s rank sum test with Holm' s correction for multiple comparisons. Number of cells are indicated above. All experiments are from triplicate experiments. **(C)** Trajectories of fluorescent spots of mCherry-KPNA3, mCherry-KPNA4, and mCherry- KPNA6 in the axons of DRG neurons (N = 64–81, number of spots from triplicate experiments. **(D)** Histogram of velocities of fluorescent spots of mCherry-KPNA3, mChery-KPNA4, and mCherry-KPNA6 in the axons of DRG neurons. Number of spots are 66–113 from 9, 11, 15 cells respectively from two time or three times experiments.

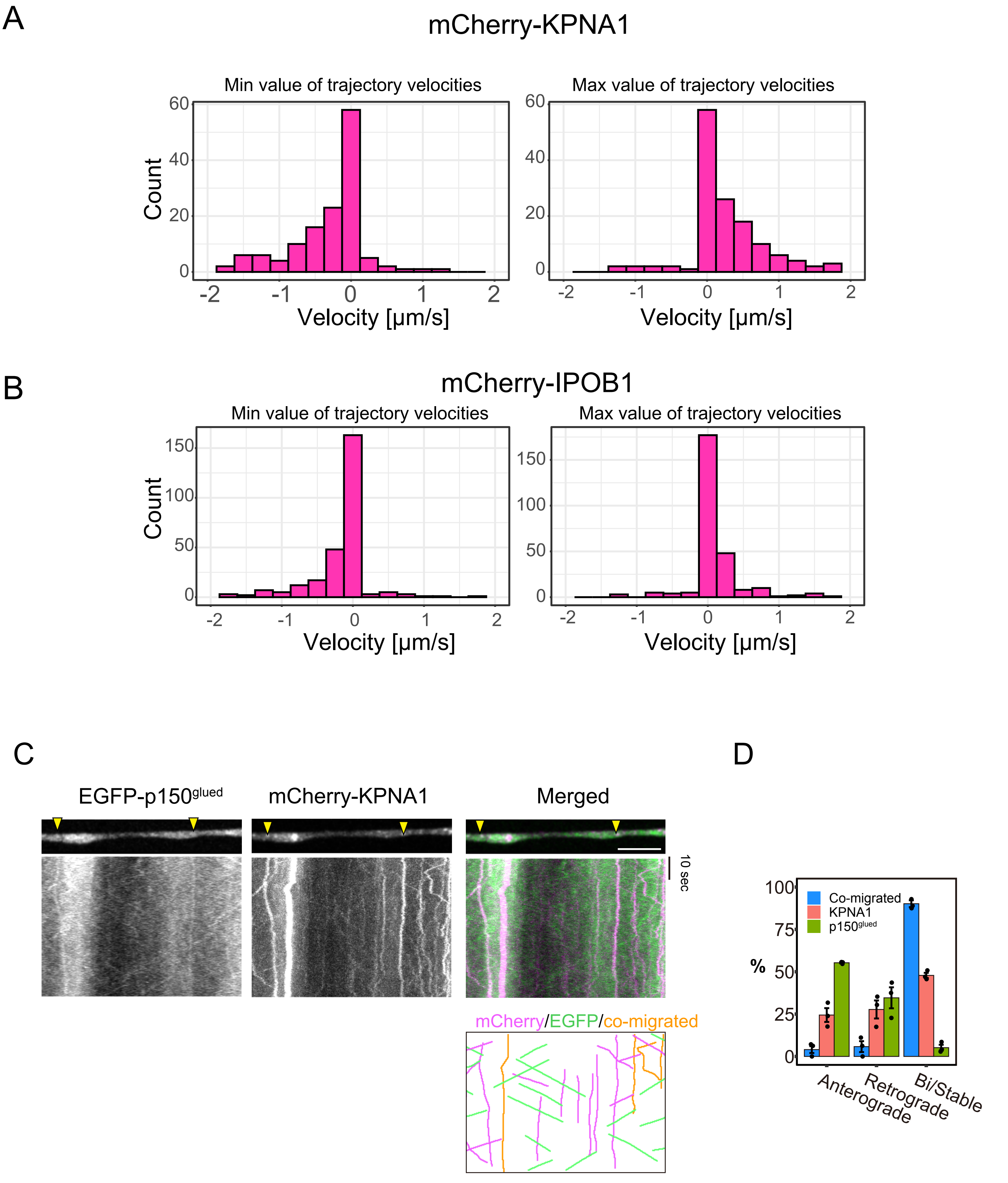

Fig. S4

(**A, B**) Histogram of minimum (left) and maximum (right) trajectory velocities of fluorescent spots for mCherry-KPNA1 (A) and mCherry-IPOB1 (B) in the axons of DRG neurons. N = 139 (A) or 274 (B) fluorescent spots from 21 cells, three biological replicates. **(C)** Axon of DRG neurons transfected with EGFP-$p150^{glued}$ and mCherry-KPNA1. Kymographs of fluorescent spots are shown at the bottom. Co-migrating traces are indicated by arrowheads. Trajectories are shown at bottom. Green trajectories represent EGFP fusion proteins. Magenta trajectories represent mCherry. Orange trajectories indicate co-migration of signals from both wavelengths. **(D)** Quantification of the motility pattern of co-migrating (blue) or independently migrating fluorescent spots. Magenta and green indicate not co-migrating KPNA1 and $p150^{glued}$, respectively. Mean percentages from three independent experiments are shown. The measurements are from 17 cells and 524 fluorescent spots from four times experiments.

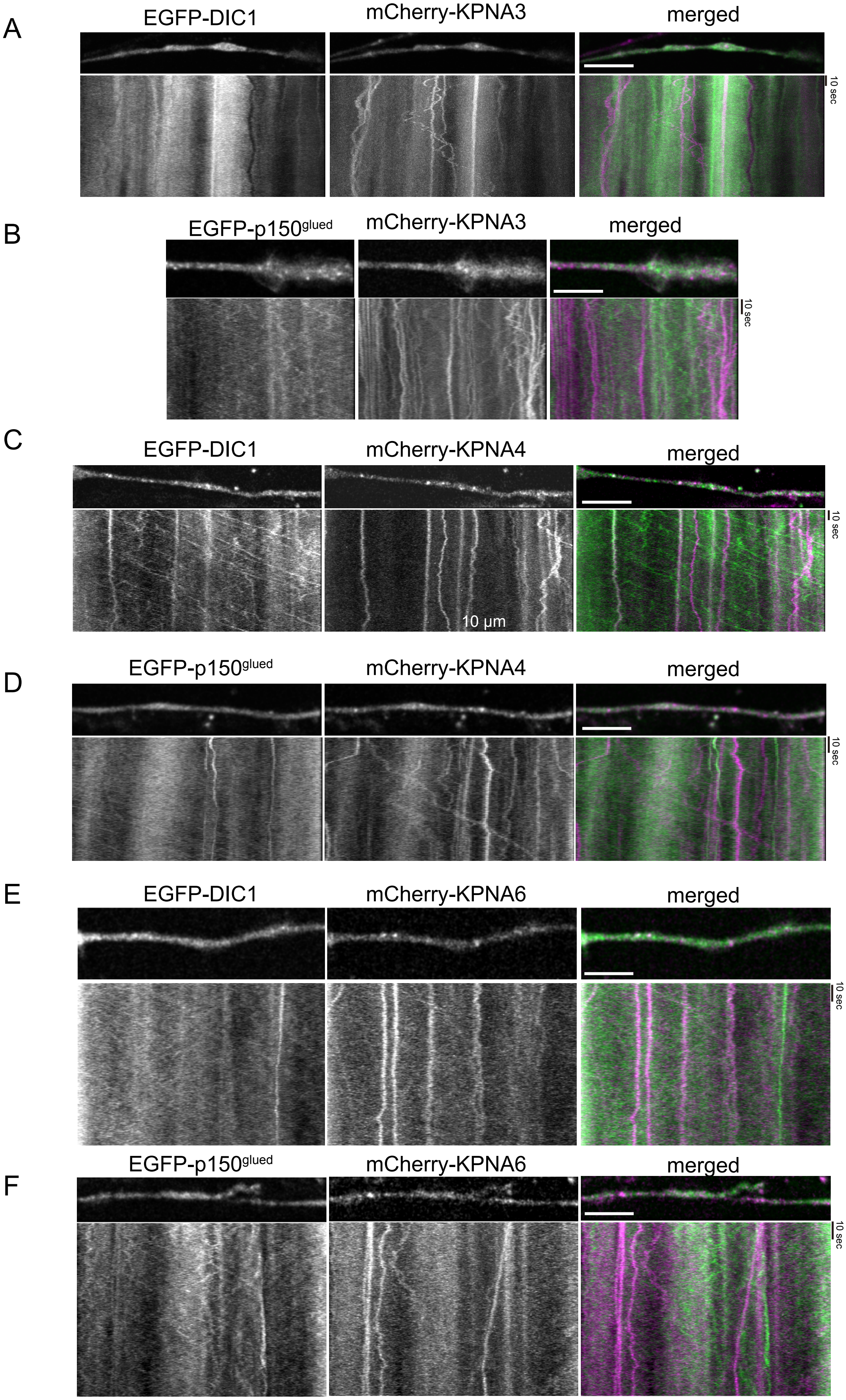

Fig. S5

The top panels show axons of DRG neurons, while the bottom panels display kymographs of fluorescent spots. Transfections include: **(A)** EGFP-DIC1 and mCherry-KPNA3, **(B)** EGFP-p150^{glued} and mCherry-KPNA3, **(C)** EGFP-DIC1 and mCherry-KPNA4, **(D)** EGFP-p150^{glued} and mCherry-KPNA4, **(E)** EGFP-DIC1 and mCherry-KPNA6, and **(F)** EGFP-p150^{glued} and mCherry- KPNA6. Scale bar: 10 µm.

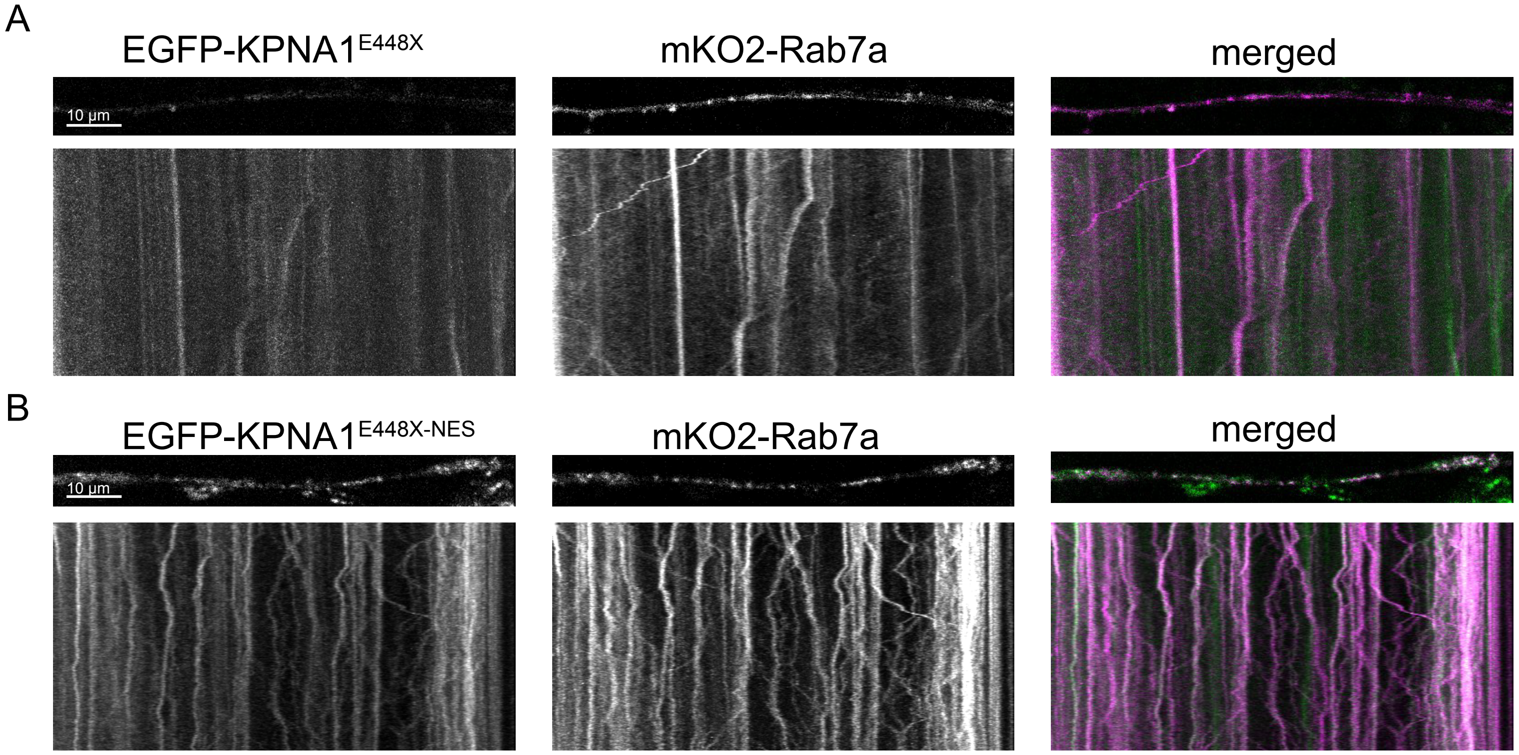

Fig. S6
(A, B). Axons of DRG neurons transfected with (A) EGFP-KPNA1 E448X and mKO2-Rab7a, or (B) EGFP-KPNA1E448X-NES. The top panels show images of axons, and the bottom panels display kymographs of fluorescent spots.
